# Confidence intervals for validation statistics with data truncation in genomic prediction

**DOI:** 10.1186/s12711-024-00883-w

**Published:** 2024-03-08

**Authors:** Matias Bermann, Andres Legarra, Alejandra Alvarez Munera, Ignacy Misztal, Daniela Lourenco

**Affiliations:** 1 Department of Animal and Dairy Science, University of Georgia, Athens, GA 30602 USA; 2 Council on Dairy Cattle Breeding (CDCB), Bowie, MD 20716 USA

## Abstract

**Background:**

Validation by data truncation is a common practice in genetic evaluations because of the interest in predicting the genetic merit of a set of young selection candidates. Two of the most used validation methods in genetic evaluations use a single data partition: predictivity or predictive ability (correlation between pre-adjusted phenotypes and estimated breeding values (EBV) divided by the square root of the heritability) and the linear regression (LR) method (comparison of “early” and “late” EBV). Both methods compare predictions with the whole dataset and a partial dataset that is obtained by removing the information related to a set of validation individuals. EBV obtained with the partial dataset are compared against adjusted phenotypes for the predictivity or EBV obtained with the whole dataset in the LR method. Confidence intervals for predictivity and the LR method can be obtained by replicating the validation for different samples (or folds), or bootstrapping. Analytical confidence intervals would be beneficial to avoid running several validations and to test the quality of the bootstrap intervals. However, analytical confidence intervals are unavailable for predictivity and the LR method.

**Results:**

We derived standard errors and Wald confidence intervals for the predictivity and statistics included in the LR method (bias, dispersion, ratio of accuracies, and reliability). The confidence intervals for the bias, dispersion, and reliability depend on the relationships and prediction error variances and covariances across the individuals in the validation set. We developed approximations for large datasets that only need the reliabilities of the individuals in the validation set. The confidence intervals for the ratio of accuracies and predictivity were obtained through the Fisher transformation. We show the adequacy of both the analytical and approximated analytical confidence intervals and compare them versus bootstrap confidence intervals using two simulated examples. The analytical confidence intervals were closer to the simulated ones for both examples. Bootstrap confidence intervals tend to be narrower than the simulated ones. The approximated analytical confidence intervals were similar to those obtained by bootstrapping.

**Conclusions:**

Estimating the sampling variation of predictivity and the statistics in the LR method without replication or bootstrap is possible for any dataset with the formulas presented in this study.

**Supplementary Information:**

The online version contains supplementary material available at 10.1186/s12711-024-00883-w.

## Background

Validation by data truncation has been proposed to validate models for genetic and genomic predictions [1]. In recent years, its popularity has increased over model-based statistics, such as the Akaike information criterion or likelihood ratio [2]. Widely used statistics for validation by data truncation are those included in the linear regression (LR) method, which compares sets of estimated breeding values (EBV) [3], and predictivity [4], the latter defined as the correlation between EBV and adjusted phenotypes, divided by the square root of the heritability. These validation statistics focus on the performance of the model to predict breeding values. Validation using these methods was done in dairy [5] and beef [6] cattle, pigs [7], chickens [8], sheep [9], goats [10], fish [11], wheat [12], and trees [13], among others. For validation in dairy cattle, using weighted averages and deregressed evaluations could be more robust than the LR method or predictivity [14]. Overall, the validation methods covered in the present study provide measures of bias and accuracy of genomic predictions. Standard errors and confidence intervals of validation statistics can be obtained by k-fold cross-validation [2]. Many studies assessed the variation of the LR method statistics by replicating the validation (e.g., [15, 16]). However, in routine genetic evaluations, k-fold validation is not useful because of population structure [1], it does not account for the reduction in variance in the selected population [3], and the interest is in predicting the genetic merit of young individuals [3]. Therefore, validation by data truncation is a common practice for routine genetic evaluations in animal and plant breeding [17–23].

In an early stage of developing the LR method, Legarra and Reverter [24] proposed calculating confidence intervals for the dispersion of the predictions (slope of the regression of true on estimated breeding value) using classical regression theory (i.e., considering ${\widehat{\mathbf{u}}}_{p}$ as fixed) [25]. However, the random and correlated nature between ${\widehat{\mathbf{u}}}_{p}$ and ${\widehat{\mathbf{u}}}_{w}$ introduces a systematic underestimation of the standard error of the dispersion. Thus, the estimated confidence intervals are narrower than the true ones.

Two methods are currently used to obtain standard errors and confidence intervals for validation by data truncation in genetic and genomic predictions. The first approach to assess the variation of validation statistics is to perform forward validations at several time points [18, 20, 23]. This practice gives an idea of the variation of the validation statistics over time. However, it cannot predict the variation of any statistics for a specific time point, and it is necessary to correct the statistics because some time periods might be more represented than others [18]. In addition, this method is computationally expensive for large datasets and involves complex manipulations of the available dataset. The second approach uses bootstrapping (sampling with replacement of the validation individuals to create pseudo-replicates of the validation dataset [17, 19, 22, 26]). Bootstrapping is attractive since it is computationally inexpensive and only requires running the validation once. To our knowledge, only Mäntysaari and Koivula [17] tested the adequacy of bootstrapping to obtain the variability of validation statistics for genomic selection, showing a good agreement with the first approach; however, this was only shown for one dairy cattle dataset. In addition, non-sampling-based, analytical confidence intervals for the LR method statistics and predictivity have not been reported, although they are of interest on their own and could simplify the process of assessing the quality of validation statistics. Therefore, the objectives of this study were to derive standard errors and analytical confidence intervals for validation by data truncation statistics used in genetic and genomic evaluations, to benchmark against their simulated sampling distributions, and to compare them against confidence intervals obtained by bootstrapping.

## Methods

In the following section, we show the general model used to derive the formulas for the confidence intervals of the different validation statistics, and a useful result for the next derivations. Then, we derive the mathematical expression for each validation statistic and suggest approximations when it is not possible to obtain the exact expressions. Finally, we describe two simulations used for testing the adequacy of the presented confidence intervals. The derivation is frequentist in nature and considers the sampling distribution of the statistics of either validation method, considering the sampling variation in the phenotypes. This is the framework used by many methods to derive confidence intervals and also by related methods such as bootstrap [27]. Indeed, Efron [28] showed that cross-validation methods with replicates have frequentists interpretations.

## Theory

For the sake of presentation, we assume a single-trait model with an additive genetic effect as the only random effect, although the results extend to other types of models:

1 $$\mathbf{y}=\mathbf{X}\mathbf{b}+\mathbf{Z}\mathbf{u}+\mathbf{e},$$

where $\mathbf{y}$ is the vector of phenotypes, $\mathbf{b}$ is the vector of fixed effects, $\mathbf{u}$ is the vector of additive genetic effects, $\mathbf{e}$ is the vector of errors, and $\mathbf{X}$ and $\mathbf{Z}$ are incidence matrices.

The validation methods in this study (LR method and predictivity) consist of splitting the data into a whole and a partial dataset, denoted with the subscripts *w* and *p*, respectively. The whole dataset has all the available phenotypes, whereas in the partial dataset the phenotypes after a given date have been removed. Then, validation methods compare EBV versus either EBV (method LR) obtained from the whole dataset, or pre-corrected phenotypes present in the “whole” but not in the “partial” dataset (predictivity). The comparison is usually for a set of individuals, named “focal”; this can be e.g. bulls acquiring progeny records in the “whole” (but not in the partial dataset) or individual pigs acquiring, say, growth records in the “whole” (but not in the partial dataset).

Predicting $\mathbf{u}$ for the validation or testing set based on the whole data $\left({\widehat{\mathbf{u}}}_{w}\right)$ requires solving the model in Eq. (1). The prediction of $\mathbf{u}$ for the validation set based on the partial data $\left({\widehat{\mathbf{u}}}_{p}\right)$ is obtained by removing the phenotypes of the individuals in the validation set before solving the model in Eq. (1). As shown in Appendix I, if $\mathbf{y}$ is assumed to follow a multivariate normal distribution and the predictions are obtained by best linear unbiased prediction in absence of selection (i.e., under random mating and random culling) [29], the joint distribution of ${\widehat{\mathbf{u}}}_{w}$ and ${\widehat{\mathbf{u}}}_{p}$ is:

2 $$\left[\begin{array}{c}{\widehat{\mathbf{u}}}_{w}\\ {\widehat{\mathbf{u}}}_{p}\end{array}\right]\sim {\text{MVN}}\left(\left[\begin{array}{c}{\mathbf{0}}\\ {\mathbf{0}}\end{array}\right],\left[\begin{array}{cc}\mathbf{G}-{\mathbf{C}}_{w}^{22}& \mathbf{G}-{\mathbf{C}}_{p}^{22}\\ \mathbf{G}-{\mathbf{C}}_{p}^{22}& \mathbf{G}-{\mathbf{C}}_{p}^{22}\end{array}\right]\right),$$

where $\mathbf{G}={\text{Var}}\left(\mathbf{u}\right)$, ${\mathbf{C}}_{w}^{22}$ is the prediction error variance of ${\widehat{\mathbf{u}}}_{w}$, and ${\mathbf{C}}_{p}^{22}$ is the prediction error variance of ${\widehat{\mathbf{u}}}_{p}$. If the predictions are obtained from mixed model equations (MME), ${\mathbf{C}}_{w}^{22}$ and ${\mathbf{C}}_{p}^{22}$ are obtained as blocks of the inverse of the MME for the animal effect. Absence of selection is assumed for simplicity and because the variances in Eq. (2) become complicated (and basically impossible in practice, as selection is not easily described algebraically) to obtain (see Appendix I), and this is a standard simplifying assumption in animal breeding applications – for instance, reliabilities are obtained from Eq. (2) or an approximation. As shown in the Appendix I, the conditional distribution of ${\widehat{\mathbf{u}}}_{w}$ given ${\widehat{\mathbf{u}}}_{p}$ is:

3 $${\widehat{\mathbf{u}}}_{w}|{\widehat{\mathbf{u}}}_{p}\sim {\text{MVN}}\left({\widehat{\mathbf{u}}}_{p},{\mathbf{C}}_{p}^{22}-{\mathbf{C}}_{w}^{22}\right).$$

Note that Eqs. (2) and (3) also hold for a subvector of ${\widehat{\mathbf{u}}}_{w}$ and ${\widehat{\mathbf{u}}}_{p}$. Thus, the following derivations hold for the entire vectors ${\widehat{\mathbf{u}}}_{w}$ and ${\widehat{\mathbf{u}}}_{p}$ (i.e., the population) as well as for a subvector of ${\widehat{\mathbf{u}}}_{w}$ and ${\widehat{\mathbf{u}}}_{p}$ (i.e., the estimated breeding values of a subset of the population).

### Bias

Legarra and Reverter [3] derived the estimate of the bias of predictions (${\mu }_{wp}$) as the difference between the averages of ${\widehat{\mathbf{u}}}_{p}$ and ${\widehat{\mathbf{u}}}_{w}$. In matrix notation:

4 $${\mu }_{wp}={{n}^{-1}{\mathbf{1}}}^{{{\prime}}}\left({\widehat{\mathbf{u}}}_{p}-{\widehat{\mathbf{u}}}_{w}\right),$$

where $n$ is the number of individuals in the testing set and **1** is a vector of ones. Because of the joint multivariate normality of ${\widehat{\mathbf{u}}}_{p}$ and ${\widehat{\mathbf{u}}}_{w}$, ${\mu }_{wp}$ is normally distributed (see p 92 in [25]). Therefore, a Wald confidence interval [30] for ${\mu }_{wp}$ can be constructed if its standard error is known. Taking the variance of Eq. (4):

5 $${\text{Var}}\left({\mu }_{wp}\right)={{n}^{-2}{\mathbf{1}}}^{{{\prime}}}{\text{Var}}\left({\widehat{\mathbf{u}}}_{p}-{\widehat{\mathbf{u}}}_{w}\right){\mathbf{1}}={{n}^{-2}{\mathbf{1}}}^{{{\prime}}}\left({\mathbf{C}}_{p}^{22}-{\mathbf{C}}_{w}^{22}\right){\mathbf{1}}.$$

The above equation is simply the difference of the averages of the prediction error variances of the predictions. Then, a confidence interval for ${\mu }_{wp}$ is:

6 $${{\text{CI}}}_{100\left(1-\alpha \right)}\left({\mu }_{wp}\right)={\mu }_{wp}\pm {z}_{1-\frac{\alpha }{2}} \sqrt{{{n}^{-2}{\mathbf{1}}}^{\mathbf{^{\prime}}}\left({\mathbf{C}}_{p}^{22}-{\mathbf{C}}_{w}^{22}\right){\mathbf{1}}},$$

where ${z}_{1-\frac{\alpha }{2}}$ is the value of the standard normal distribution quantile function for the confidence level $1-\frac{\alpha }{2}$. For large datasets, it is computationally unfeasible to obtain ${\mathbf{C}}_{p}^{22}$ and ${\mathbf{C}}_{w}^{22}$. In that situation, we can simplify Eq. (6), assuming that animals are non-inbred and mostly unrelated such that the off-diagonal elements of $\mathbf{G}$, ${\mathbf{C}}_{w}^{22}$, and ${\mathbf{C}}_{p}^{22}$ can be safely ignored. Thus, ${\mathbf{C}}_{p}^{22}-{\mathbf{C}}_{w}^{22}\approx {\mathbf{R}}_{w}-{\mathbf{R}}_{p}$, where ${\mathbf{R}}_{w}$ and ${\mathbf{R}}_{p}$ are diagonal matrices of genomic (G)EBV’s reliabilities in the whole and partial datasets, respectively. Letting ${\sigma }_{g}^{2}$ be the genetic variance, ${\text{Var}}\left({\mu }_{wp}\right)\approx \frac{{\sigma }_{g}^{2}}{n}\left({\overline{rel} }_{w}-{\overline{rel} }_{p}\right)$ and an approximate confidence interval for ${\mu }_{wp}$ is:

7 $${{\text{CI}}}_{100\left(1-\alpha \right)}\left({\mu }_{wp}\right)\approx {\mu }_{wp}\pm {z}_{1-\frac{\alpha }{2}} \sqrt{\frac{{\sigma }_{g}^{2}}{n}\left({\overline{rel} }_{w}-{\overline{rel} }_{p}\right)}.$$

### Dispersion

The regression coefficient of ${\widehat{\mathbf{u}}}_{w}$ on ${\widehat{\mathbf{u}}}_{p}$ $({b}_{wp})$ quantifies the dispersion of the predictions with partial data. If there is no under/over dispersion, the expected value of ${b}_{wp}$ is equal to 1. The mathematical expression for ${b}_{wp}$ is:

8 $${b}_{wp}=\frac{cov\left({\widehat{\mathbf{u}}}_{w},{\widehat{\mathbf{u}}}_{p}\right)}{var\left({\widehat{\mathbf{u}}}_{p}\right)}=\frac{{\widehat{\mathbf{u}}}_{w}^{\mathbf{^{\prime}}}\mathbf{S} {\widehat{\mathbf{u}}}_{p}}{{\widehat{\mathbf{u}}}_{p}^{\mathbf{^{\prime}}}\mathbf{S} {\widehat{\mathbf{u}}}_{p}},$$

where $cov$ and $var$ are the sample covariance and variance, respectively, and $\mathbf{S}=\mathbf{I}-{n}^{-1}{\mathbf{11}}^{\prime}$. A Wald confidence interval for ${b}_{wp}$ can be constructed because ${b}_{wp}$ is asymptotically normal when the number of focal individuals in the validation set increases (see p. 249 in [25]). By the law of the total variance (see p. 167 in [31]):

9 $${\text{Var}}\left({b}_{wp}\right)={\text{E}}\left[{\text{Var}}\left({b}_{wp}|{\widehat{\mathbf{u}}}_{p}\right)\right]+{\text{Var}}\left[{\text{E}}\left({b}_{wp}|{\widehat{\mathbf{u}}}_{p}\right)\right].$$

For the first term in the right-hand side, we have:

10 $${\text{E}}\left[{\text{Var}}\left({b}_{wp}|{\widehat{\mathbf{u}}}_{p}\right)\right]={\text{E}}\left[\frac{{\widehat{\mathbf{u}}}_{p}^{\mathrm{^{\prime}}}\mathbf{S}\,{\text{Var}}\left({\widehat{\mathbf{u}}}_{w}|{\widehat{\mathbf{u}}}_{p}\right)\mathbf{S}\,{\widehat{\mathbf{u}}}_{p}}{{\left({\widehat{\mathbf{u}}}_{p}^{\mathrm{^{\prime}}}\mathbf{S}\,{\widehat{\mathbf{u}}}_{p}\right)}^{2}}\right]={\text{E}}\left[\frac{{\widehat{\mathbf{u}}}_{p}^{\mathrm{^{\prime}}}\mathbf{S}\left({\mathbf{C}}_{p}^{22}-{\mathbf{C}}_{w}^{22}\right)\mathbf{S}\,{\widehat{\mathbf{u}}}_{p}}{{\left({\widehat{\mathbf{u}}}_{p}^{\mathrm{^{\prime}}}\mathbf{S}\,{\widehat{\mathbf{u}}}_{p}\right)}^{2}}\right].$$

Using a first-order Taylor approximation:

11 $${\text{E}}\left[\frac{{\widehat{\mathbf{u}}}_{p}^{\mathrm{^{\prime}}}\mathbf{S}\left({\mathbf{C}}_{p}^{22}-{\mathbf{C}}_{w}^{22}\right)\mathbf{S}\,{\widehat{\mathbf{u}}}_{p}}{{\left({\widehat{\mathbf{u}}}_{p}^{\mathrm{^{\prime}}}\mathbf{S}\,{\widehat{\mathbf{u}}}_{p}\right)}^{2}}\right]\approx \frac{{\text{E}}\left[{\widehat{\mathbf{u}}}_{p}^{\mathrm{^{\prime}}}\mathbf{S}\left({\mathbf{C}}_{p}^{22}-{\mathbf{C}}_{w}^{22}\right)\mathbf{S}\,{\widehat{\mathbf{u}}}_{p}\right]}{{\text{E}}\left[{\left({\widehat{\mathbf{u}}}_{p}^{\mathrm{^{\prime}}}\mathbf{S}\,{\widehat{\mathbf{u}}}_{p}\right)}^{2}\right]}.$$

By the expectation of quadratic forms [32] and the zero expectation of ${\widehat{\mathbf{u}}}_{p}$ and ${\widehat{\mathbf{u}}}_{w}$, the numerator of the right-hand side in Eq. (11) is ${\text{E}}\left[{\widehat{\mathbf{u}}}_{p}^{\prime}\mathbf{S}\left({\mathbf{C}}_{p}^{22}-{\mathbf{C}}_{w}^{22}\right)\mathbf{S} {\widehat{\mathbf{u}}}_{p}\right]={\text{tr}}\left(\mathbf{S}\left({\mathbf{C}}_{p}^{22}-{\mathbf{C}}_{w}^{22}\right)\mathbf{S}\left(\mathbf{G}-{\mathbf{C}}_{p}^{22}\right)\right)$. For the denominator, we have ${\text{E}}\left[{\left({\widehat{\mathbf{u}}}_{p}^{\prime}\mathbf{S} {\widehat{\mathbf{u}}}_{p}\right)}^{2}\right]={\text{Var}}\left({\widehat{\mathbf{u}}}_{p}^{\prime}\mathbf{S} {\widehat{\mathbf{u}}}_{p}\right)+{\text{E}}{\left[{\widehat{\mathbf{u}}}_{p}^{\prime}\mathbf{S} {\widehat{\mathbf{u}}}_{p}\right]}^{2}=2\,{\text{tr}}\left(\mathbf{S}\left(\mathbf{G}-{\mathbf{C}}_{p}^{22}\right)\mathbf{S}\left(\mathbf{G}-{\mathbf{C}}_{p}^{22}\right)\right)+{\text{tr}}{\left(\mathbf{S}\left(\mathbf{G}-{\mathbf{C}}_{p}^{22}\right)\right)}^{2}$. Thus:

12 $${\text{E}}\left[{\text{Var}}\left({b}_{wp}|{\widehat{\mathbf{u}}}_{p}\right)\right]\approx \frac{{\text{tr}}\left(\mathbf{S} \left({\mathbf{C}}_{p}^{22}-{\mathbf{C}}_{w}^{22}\right) \mathbf{S}\left(\mathbf{G}-{\mathbf{C}}_{p}^{22}\right)\right)}{2\,\mathrm{ tr}\left(\mathbf{S} \left(\mathbf{G}-{\mathbf{C}}_{p}^{22}\right) \mathbf{S}\left(\mathbf{G}-{\mathbf{C}}_{p}^{22}\right)\right)+{\text{tr}}{\left(\mathbf{S} \left(\mathbf{G}-{\mathbf{C}}_{p}^{22}\right) \right)}^{2}}.$$

For the second term in the right-hand side of Eq. (9):

13 $${\text{Var}}\left[{\text{E}}\left({b}_{wp}|{\widehat{\mathbf{u}}}_{p}\right)\right]={\text{Var}}\left(\frac{{\widehat{\mathbf{u}}}_{p}^{\mathrm{^{\prime}}}\mathbf{S}\,{\text{E}}\left({\widehat{\mathbf{u}}}_{w}|{\widehat{\mathbf{u}}}_{p}\right)}{{\widehat{\mathbf{u}}}_{p}^{\mathrm{^{\prime}}}\mathbf{S}\,{\widehat{\mathbf{u}}}_{p}}\right)={\text{Var}}\left(\frac{{\widehat{\mathbf{u}}}_{p}^{\mathrm{^{\prime}}}\mathbf{S}\,{\widehat{\mathbf{u}}}_{p}}{{\widehat{\mathbf{u}}}_{p}^{\mathrm{^{\prime}}}\mathbf{S} \,{\widehat{\mathbf{u}}}_{p}}\right)=0.$$

Therefore, the variance of ${b}_{wp}$ is:

14 $${\text{Var}}\left({b}_{wp}\right)\approx \frac{{\text{tr}}\left(\mathbf{S} \left({\mathbf{C}}_{p}^{22}-{\mathbf{C}}_{w}^{22}\right) \mathbf{S}\left(\mathbf{G}-{\mathbf{C}}_{p}^{22}\right)\right)}{2\,\mathrm{ tr}\left(\mathbf{S} \left(\mathbf{G}-{\mathbf{C}}_{p}^{22}\right) \mathbf{S}\left(\mathbf{G}-{\mathbf{C}}_{p}^{22}\right)\right)+{\text{tr}}{\left(\mathbf{S} \left(\mathbf{G}-{\mathbf{C}}_{p}^{22}\right) \right)}^{2}},$$

and the Wald confidence interval for ${b}_{wp}$ is:

15 $${{\text{CI}}}_{100\left(1-\alpha \right)}\left({b}_{wp}\right)={b}_{wp}\pm {z}_{1-\frac{\alpha }{2}} \sqrt{\frac{{\text{tr}}\left(\mathbf{S}\left({\mathbf{C}}_{p}^{22}-{\mathbf{C}}_{w}^{22}\right)\mathbf{S}\left(\mathbf{G}-{\mathbf{C}}_{p}^{22}\right)\right)}{2\,\mathrm{ tr}\left(\mathbf{S}\left(\mathbf{G}-{\mathbf{C}}_{p}^{22}\right)\mathbf{S}\left(\mathbf{G}-{\mathbf{C}}_{p}^{22}\right)\right)+{\text{tr}}{\left(\mathbf{S}\left(\mathbf{G}-{\mathbf{C}}_{p}^{22}\right)\right)}^{2}}}.$$

By making similar assumptions as for the estimator of the bias, $\mathbf{G}-{\mathbf{C}}_{p}^{22}\approx {\mathbf{R}}_{p}$. Then, ${\text{tr}}\left(\mathbf{S}\left({\mathbf{C}}_{p}^{22}-{\mathbf{C}}_{w}^{22}\right)\mathbf{S}\left(\mathbf{G}-{\mathbf{C}}_{p}^{22}\right)\right)\approx {\sigma }_{g}^{4}{\sum }_{i=1}^{n}\left(re{l}_{{w}_{i}}-re{l}_{{p}_{i}}\right)re{l}_{{p}_{i}}$, ${\text{tr}}\left(\mathbf{S}\left(\mathbf{G}-{\mathbf{C}}_{p}^{22}\right)\mathbf{S}\left(\mathbf{G}-{\mathbf{C}}_{p}^{22}\right)\right)\approx {\sigma }_{g}^{4}{\sum }_{i=1}^{n}re{l}_{{p}_{i}}^{2}$, and ${\text{tr}}{\left(\mathbf{S}\left(\mathbf{G}-{\mathbf{C}}_{p}^{22}\right)\right)}^{2}\approx {\sigma }_{g}^{4}{\left({\sum }_{i=1}^{n}re{l}_{{p}_{i}}\right)}^{2}$, which results in:

16 $${\text{Var}}\left({b}_{wp}\right)\approx \frac{{\sum }_{i=1}^{n}\left(re{l}_{{w}_{i}}-re{l}_{{p}_{i}}\right) re{l}_{{p}_{i}}}{2 {\sum }_{i=1}^{n}re{l}_{{p}_{i}}^{2}+{\left({\sum }_{i=1}^{n}re{l}_{{p}_{i}}\right)}^{2}},$$

from which an approximate confidence interval for ${b}_{wp}$ can be constructed. Assuming that the increase in reliability from the partial to the whole dataset is constant among the validation animals, $\frac{re{l}_{{w}_{i}}}{re{l}_{{p}_{i}}}=c$ (which is always higher than 1). Then, an approximate confidence interval for ${b}_{wp}$ is:

17 $${{\text{CI}}}_{100\left(1-\alpha \right)}\left({b}_{wp}\right)\approx {b}_{wp}\pm {z}_{1-\frac{\alpha }{2}} \sqrt{\frac{\left(c-1\right)\left(Var\left(re{l}_{p}\right)+{\overline{rel} }_{p}^{2}\right)}{2 \left(Var\left(re{l}_{p}\right)+{\overline{rel} }_{p}^{2}\right)+n {\overline{rel} }_{p}^{2}}}.$$

### Ratio of accuracies

The Pearson correlation coefficient between ${\widehat{\mathbf{u}}}_{p}$ and ${\widehat{\mathbf{u}}}_{w}$ $\left({\rho }_{wp}\right)$ has an expected value equal to the ratio of accuracies obtained with the partial and the whole dataset. The formula for ${\rho }_{wp}$ is:

18 $${\rho }_{wp}=\frac{cov\left({\widehat{\mathbf{u}}}_{w},{\widehat{\mathbf{u}}}_{p}\right)}{\sqrt{var\left({\widehat{\mathbf{u}}}_{w}\right) var\left({\widehat{\mathbf{u}}}_{p}\right)}}.$$

In principle, a confidence interval can be obtained that involves explicitly elements in Eq. (2); however, this yielded inelegant expressions that were unusable in practice (see Appendix II). We propose to use a confidence interval for ${\rho }_{wp}$ using the Fisher transformation [25] and (see p. 261 in [29]). The inverse hyperbolic tangent of a correlation coefficient $r$ $\left({{\text{tanh}}}^{-1}\left(r\right)=\frac{1}{2}{\text{log}}\left(\frac{1+r}{1-r}\right)\right)$ follows approximately a normal distribution with a standard error equal to $\frac{1}{\sqrt{n-3}}$ assuming that the samples are identically and independently distributed (which is not the case for genetic evaluations). Thus:

19 $${{\text{CI}}}_{100\left(1-\alpha \right)}\left({{\text{tanh}}}^{-1}\left({\rho }_{wp}\right)\right)={{\text{tanh}}}^{-1}\left({\rho }_{wp}\right)\pm {z}_{1-\frac{\alpha }{2}}\frac{1}{\sqrt{n-3}}.$$

To obtain a confidence interval for ${\rho }_{wp}$, we apply the hyperbolic tangent and get:

20 $${{\text{CI}}}_{100\left(1-\alpha \right)}\left({\rho }_{wp}\right)={\text{tanh}}\left({{\text{tanh}}}^{-1}\left({\rho }_{wp}\right)\pm {z}_{1-\frac{\alpha }{2}}\frac{1}{\sqrt{n-3}}\right),$$

where $${\text{tanh}}\left(x\right)=\frac{{e}^{2x}-1}{{e}^{2x}+1}$$.

This can be computed for any dataset size but note that this confidence interval is not symmetric around ${\rho }_{wp}$.

### Reliability

The reliability of the EBV is defined as the square of the correlation between the true and estimated breeding values. Legarra and Reverter [3] and Macedo et al. [29] proposed that the reliability be estimated as the ratio between the sample covariance of ${\widehat{\mathbf{u}}}_{p}$ and ${\widehat{\mathbf{u}}}_{w}$ and the genetic variance of the validation set $\left({\sigma }_{{g}_{i}}^{2}\right)$. This variance must account for selection and can be approximated using averages of additive relationships among validation animals, which accounts for e.g. few families of large sibships, or calculated with the method of Sorensen et al. [33] which correctly accounts for selection. We will assume this variance as known. The estimator of the reliability has the following expression:

21 $${\rho }_{co{v}_{wp}}^{2}=\frac{{\widehat{\mathbf{u}}}_{w}^{\mathrm{^{\prime}}}\mathbf{S}\,{\widehat{\mathbf{u}}}_{p}}{n {\sigma }_{{g}_{i}}^{2}}.$$

Although ${\rho }_{co{v}_{wp}}^{2}$ is not normally distributed, we assume that, for large sample sizes, its distribution is approximately normal. Taking the variance of ${\rho }_{co{v}_{wp}}^{2}$ gives:

22 $${\text{Var}}\left({\rho }_{co{v}_{wp}}^{2}\right)=\frac{{\text{Var}}\left({\widehat{\mathbf{u}}}_{w}^{\mathrm{^{\prime}}}\mathbf{S}\,{\widehat{\mathbf{u}}}_{p}\right)}{{\left(n {\sigma }_{{g}_{i}}^{2}\right)}^{2}}.$$

As in Eq. (9), we apply the law of total variance for the numerator of the right-hand side in Eq. (22):

23 $${\text{Var}}\left({\widehat{\mathbf{u}}}_{w}^{\mathrm{^{\prime}}}\mathbf{S}\,{\widehat{\mathbf{u}}}_{p}\right)={\text{E}}\left[{\text{Var}}\left({\widehat{\mathbf{u}}}_{w}^{\mathrm{^{\prime}}}\mathbf{S}\,{\widehat{\mathbf{u}}}_{p}|{\widehat{\mathbf{u}}}_{p} \right)\right]+{\text{Var}}\left({\text{E}}\left[{\widehat{\mathbf{u}}}_{w}^{\mathrm{^{\prime}}}\mathbf{S}\,{\widehat{\mathbf{u}}}_{p}|{\widehat{\mathbf{u}}}_{p}\right]\right).$$

Following similar arguments as for Eqs. (10) and (11), the first term is equal to ${\text{tr}}\left(\mathbf{S}\left({\mathbf{C}}_{p}^{22}-{\mathbf{C}}_{w}^{22}\right)\mathbf{S}\left(\mathbf{G}-{\mathbf{C}}_{p}^{22}\right)\right)$, whereas the second term is equal to $2\,{\text{tr}}\left(\mathbf{S}\left(\mathbf{G}-{\mathbf{C}}_{p}^{22}\right)\mathbf{S}\left(\mathbf{G}-{\mathbf{C}}_{p}^{22}\right)\right)$. Therefore:

24 $${\text{Var}}\left({\rho }_{co{v}_{wp}}^{2}\right)=\frac{{\text{tr}}\left(\mathbf{S}\left({\mathbf{C}}_{p}^{22}-{\mathbf{C}}_{w}^{22}\right)\mathbf{S}\left(\mathbf{G}-{\mathbf{C}}_{p}^{22}\right)\right)+2\,\mathrm{ tr}\left(\mathbf{S}\left(\mathbf{G}-{\mathbf{C}}_{p}^{22}\right)\mathbf{S}\left(\mathbf{G}-{\mathbf{C}}_{p}^{22}\right)\right)}{{\left(n {\sigma }_{{g}_{i}}^{2}\right)}^{2}}.$$

Finally, a confidence interval for ${\rho }_{co{v}_{wp}}^{2}$ is constructed as:

25 $${{\text{CI}}}_{100\left(1-\alpha \right)}\left({\rho }_{co{v}_{wp}}^{2}\right)={\rho }_{co{v}_{wp}}^{2}\pm {z}_{1-\frac{\alpha }{2}} \frac{\sqrt{{\text{tr}}\left(\mathbf{S}\left({\mathbf{C}}_{p}^{22}-{\mathbf{C}}_{w}^{22}\right)\mathbf{S}\left(\mathbf{G}-{\mathbf{C}}_{p}^{22}\right)\right)+2\,\mathrm{ tr}\left(\mathbf{S}\left(\mathbf{G}-{\mathbf{C}}_{p}^{22}\right)\mathbf{S}\left(\mathbf{G}-{\mathbf{C}}_{p}^{22}\right)\right)}}{n {\sigma }_{{g}_{i}}^{2}}.$$

Following the same assumptions as for the bias and dispersion parameter leads to ${\text{Var}}\left({\rho }_{co{v}_{wp}}^{2}\right)\approx \frac{{\upsigma }_{{\text{g}}}^{4}}{{\left(n {\sigma }_{{g}_{i}}^{2}\right)}^{2}}{\sum }_{i=1}^{n}\left(re{l}_{{w}_{i}}+re{l}_{{p}_{i}}\right)re{l}_{{p}_{i}}$. Assuming that the increase in reliability from the partial to the whole dataset is constant among the validation animals, that is, $\frac{re{l}_{{w}_{i}}}{re{l}_{{p}_{i}}}=c$, gives ${\text{Var}}\left({\rho }_{co{v}_{wp}}^{2}\right)\approx \frac{\left(1+c\right){\upsigma }_{{\text{g}}}^{4}}{n{\sigma }_{{g}_{i}}^{4}}\left(Var\left(re{l}_{p}\right)+{\overline{rel} }_{p}^{2}\right)$. Thus, an approximate confidence interval for ${\rho }_{co{v}_{wp}}^{2}$ is:

26 $${{\text{CI}}}_{100\left(1-\alpha \right)}\left({\rho }_{co{v}_{wp}}^{2}\right)\approx {\rho }_{co{v}_{wp}}^{2}\pm {z}_{1-\frac{\alpha }{2}} \sqrt{\frac{\left(1+c\right){\upsigma }_{{\text{g}}}^{4}}{n{\sigma }_{{g}_{i}}^{4}}\left(Var\left(re{l}_{p}\right)+{\overline{rel} }_{p}^{2}\right)}.$$

### Predictivity

The ratio between the correlation of ${\widehat{\mathbf{u}}}_{p}$ and the phenotypes of the validation set adjusted for fixed effects $\left({\mathbf{y}}^{*}\right)$ and the square root of the heritability $\left(h\right)$ is an estimate of the correlation between estimated and true breeding values [4]. This statistic is sometimes called predictivity $\left({\rho }_{{\mathbf{y}}^{*},{\widehat{\mathbf{u}}}_{p}}\right)$ and has the following mathematical expression:

27 $${\rho }_{{\mathbf{y}}^{*},{\widehat{\mathbf{u}}}_{p}}=\frac{1}{h}\frac{cov\left({\mathbf{y}}^{*},{\widehat{\mathbf{u}}}_{p}\right)}{\sqrt{var\left({\mathbf{y}}^{*}\right) var\left({\widehat{\mathbf{u}}}_{p}\right)}}.$$

As with the ratio of accuracies, the Fisher transformation can be used to obtain the following confidence interval for ${\rho }_{{\mathbf{y}}^{*},{\widehat{\mathbf{u}}}_{p}}$:

28 $${{\text{CI}}}_{100\left(1-\alpha \right)}\left({\rho }_{{\mathbf{y}}^{*},{\widehat{\mathbf{u}}}_{p}}\right)=\frac{1}{h}{\text{tanh}}\left(h{\text{atanh}}\left({\rho }_{{\mathbf{y}}^{*},{\widehat{\mathbf{u}}}_{p}}\right)\pm {z}_{1-\frac{\alpha }{2}}\frac{1}{\sqrt{n-3}}\right).$$

This can be computed for any dataset size.

## Simulations

We tested the adequacy of our analytical [(Eqs. (6), (15), (20), (25), and (28)] and approximated analytical [Eqs. (7), (17), and (26)] confidence intervals using two simulated examples. In both, we obtained the empirical distribution of the validation statistics by replicating the simulation. Then, we compared the standard error and 95% confidence interval of that sampling distribution (i.e., True) versus confidence intervals obtained with the formulas presented in the previous section (i.e., Analytical or Approximated), and by bootstrapping. The confidence intervals with bootstrap were obtained by sampling with replacement of the validation set, replicated 10,000 times.

### ***Example 1***

The first dataset was created using a publicly available pedigree created by Yutaka Masuda (https://github.com/masuday/data/blob/master/tutorial/rawfiles/rawped). The pedigree had 11 generations without selection (i.e., random mating and random culling) and 4641 individuals. Single-trait models with generation as a fixed effect $\left(\mathbf{b}\right)$ and additive genetic effect $\left(\mathbf{u}\right)$ as a random effect were simulated for different heritabilities $\left({h}^{2}\right)$ and proportions $\left(prop\right)$ of animals with phenotypes in the population. In total, a grid of 81 scenarios corresponding to ${h}^{2}=\left\{0.1, 0.2, 0.3, 0.4, 0.5, 0.6, 0.7, 0.8, 0.9\right\}$ and $prop=\left\{0.1, 0.2, 0.3, 0.4, 0.5, 0.6, 0.7, 0.8, 0.9\right\}$ was evaluated. Each scenario was replicated 50 times by sampling the vector of phenotypes from a multivariate normal distribution with mean $\mathbf{X}\mathbf{b}$ (**b** is fixed across replicates) and variance $\mathbf{Z}\mathbf{A}{\mathbf{Z}}^{\prime}{\sigma }_{a}^{2}+\mathbf{I}{\sigma }_{e}^{2}$, where $\mathbf{A}$ is the numerator relationship matrix [34], ${\sigma }_{a}^{2}=1$ and ${\sigma }_{e}^{2}=\frac{1}{{h}^{2}}-1$. The validation set was composed of phenotyped animals from the most recent generation. The number of animals in the validation set was constant among heritabilities, and for each ${prop}_{i}$ was equal to 44, 74, 119, 149, 188, 234, 274, 318, and 362, respectively. All the computations were done in Julia [35].

### ***Example 2***

For the second example, we replicated the simulation of Vitezica et al. [36], which consists of a dairy cattle selection scheme with single-step genomic best linear unbiased predictor [37–39]. In each replicate, the partial dataset was created by removing the phenotypes of the cows in the most recent generation. Two validation sets were created: the cows for which the phenotypes were removed and the sires of those cows. The number of cows in the validation set was equal to 1300, whereas the number of bulls was 200. The simulation was replicated 30 times. Estimated breeding values and exact prediction error variances were obtained with the BLUPF90 + software [40]. Prediction error variances were obtained using sparse inversion techniques, which calculates the elements in the inverse corresponding to the non-zero elements of the original matrix [41, 42]. All the other analyses were done using Julia [35].

## Results

Table 1 shows the average squared difference between the estimated and true values of the variance and 95% confidence interval bounds for Example 1. Average squared differences grouped by different $prop$ and ${h}^{2}$ are reported (see Additional file 1: Tables S1 and S2). The analytical confidence intervals and variances were closer to the true simulated values than those obtained by bootstrapping. The confidence intervals obtained by bootstrapping were very similar to the approximated analytical confidence intervals.

**Table 1:** Mean squared differences between estimated and true variance, lower bound of the 95% confidence interval (lCI), and upper bound of the 95% confidence interval (uCI), averaged over all levels of heritability and proportion of animals with records for the different validation statistics for Example 1

		Var	lCI	uCI
Bias	Analytical	4.07E−08	8.88E−04	1.07E−03
	Approximated	1.03E−05	3.71E−03	5.39E−03
	Bootstrap	1.38E−05	2.64E−03	4.04E−03
Dispersion	Analytical	4.27E−05	4.07E−02	7.92E−02
	Approximated	1.58E−04	4.89E−02	7.05E−02
	Bootstrap	2.34E−04	6.71E−02	8.01E−02
Ratio of accuracies	Analytical	–	1.38E−02	8.41E−03
	Bootstrap	3.46E−05	1.64E−02	9.95E−03
Predictivity	Analytical	–	4.01E−02	4.02E−02
	Bootstrap	2.39E−04	5.58E−02	3.42E−02
Reliability	Analytical	1.74E−06	1.39E−03	5.81E−03
	Approximated	2.74E−05	5.81E−03	1.66E−02
	Bootstrap	3.48E−05	4.58E−03	2.46E−02

**Fig. 1:**
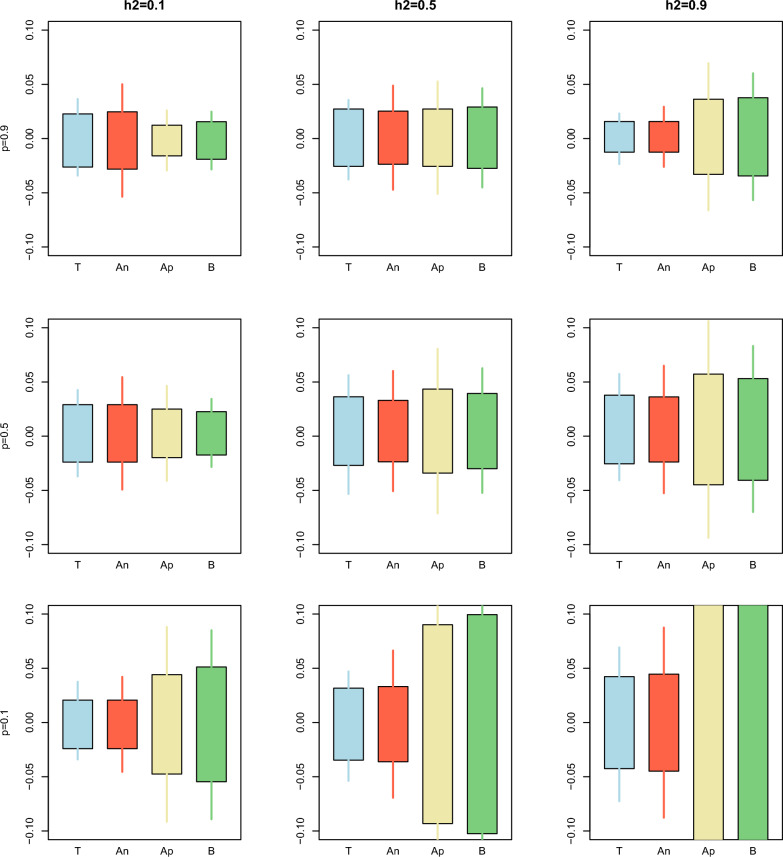
Comparison between the true (T), analytical (An), approximated (Ap), and bootstrap (B) standard error and confidence interval for the estimator of the bias over different combinations of heritability $\left({h}^{2}\right)$ and proportion of animals with records $\left(p\right)$ for Example 1. The length of the box indicates the magnitude of the standard error with respect to the mean of the bias over the replicates. The length of the whiskers indicates the length of the 95% confidence interval

**Fig. 2:**
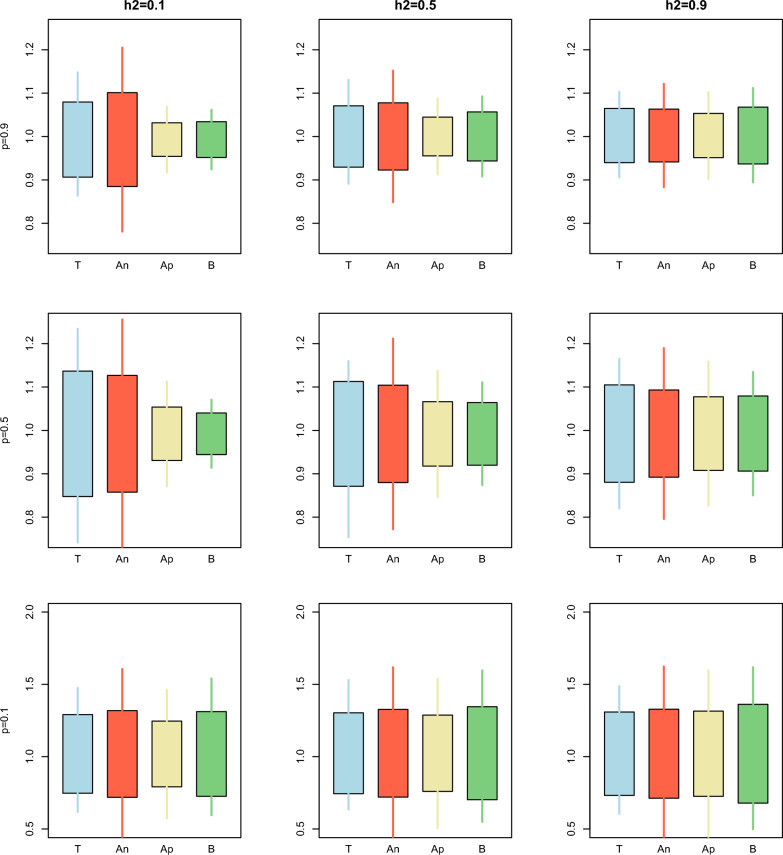
Comparison between the true (T), analytical (An), approximated (Ap), and bootstrap (B) standard error and confidence interval for the estimator of the dispersion over different combinations of heritability $\left({h}^{2}\right)$ and proportion of animals with records $\left(p\right)$ for Example 1. The length of the box indicates the magnitude of the standard error with respect to the mean of the dispersion over the replicates. The length of the whiskers indicates the length of the 95% confidence interval

**Fig. 3:**
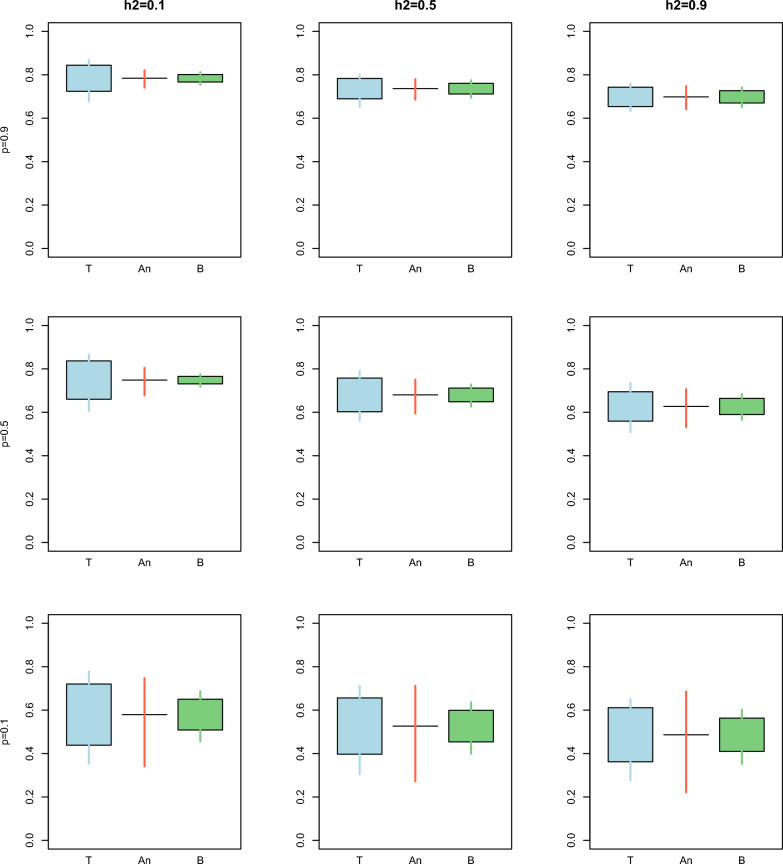
Comparison between the true (T), analytical (An), and bootstrap (B) standard error^1^ and confidence interval for the estimator of the ratio of accuracies over different combinations of heritability $\left({h}^{2}\right)$ and proportion of animals with records $\left(p\right)$ for Example 1. The length of the box indicates the magnitude of the standard error with respect to the mean. The length of the whiskers indicates the length of the 95% confidence interval. ^1^Standard errors were not available for the analytical confidence interval

**Fig. 4:**
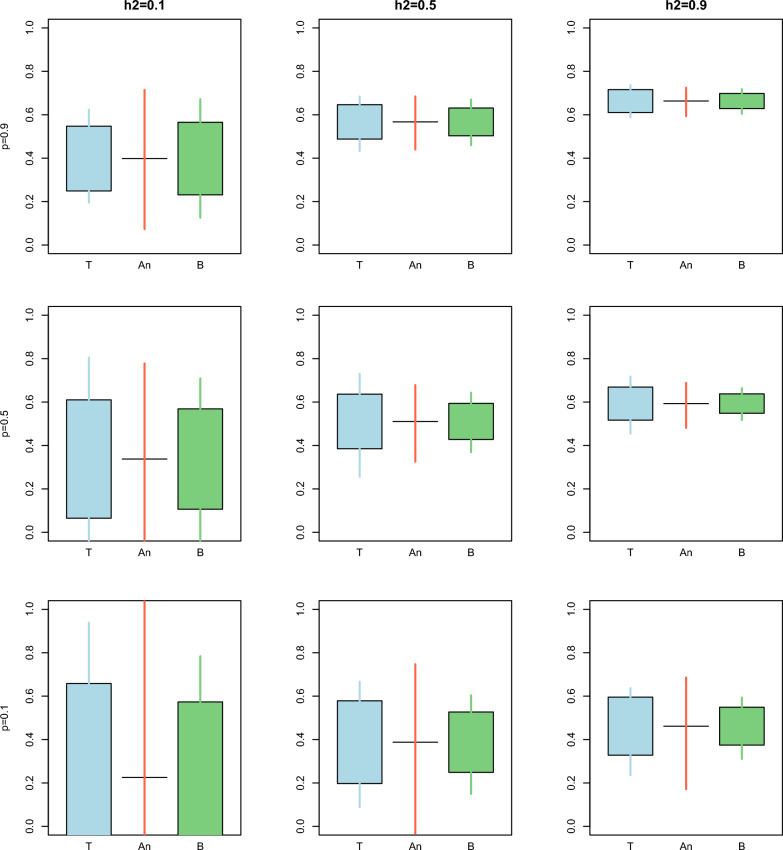
Comparison between the true (T), analytical (An), and bootstrap (B) standard error^1^ and confidence interval for the predictivity over different combinations of heritability $\left({h}^{2}\right)$ and proportion of animals with records $\left(p\right)$ for Example 1. The length of the box indicates the magnitude of the standard error with respect to the mean. The length of the whiskers indicates the length of the 95% confidence interval. ^1^Standard errors were not available for the analytical confidence interval

**Fig. 5:**
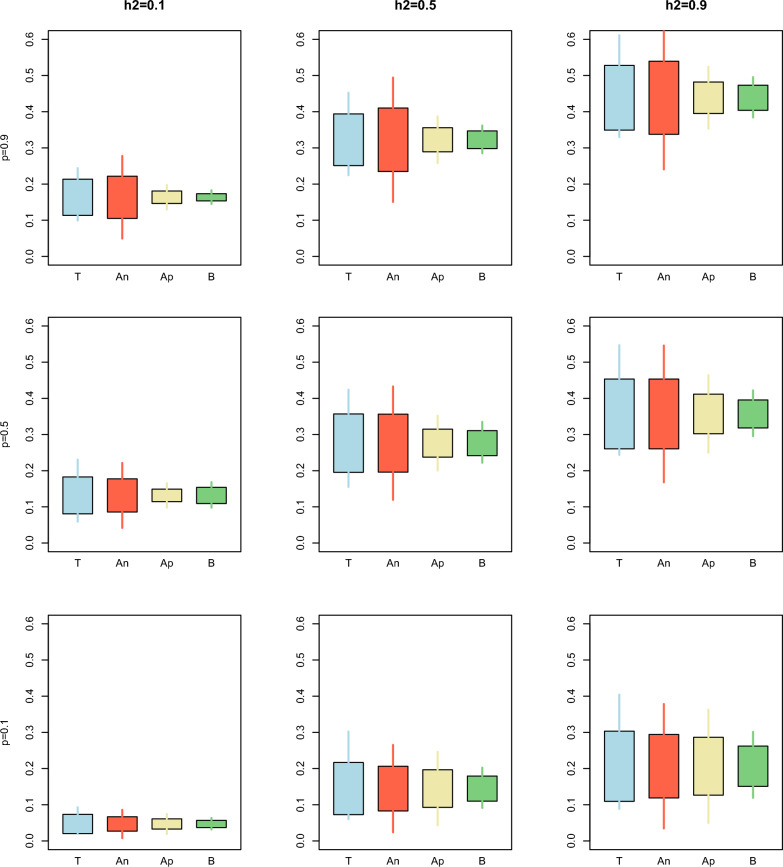
Comparison between the true (T), analytical (An), approximated (Ap), and bootstrap (B) standard error and confidence interval for the estimator of the reliability over different combinations of heritability $\left({h}^{2}\right)$ and proportion of animals with records $\left(p\right)$ for Example 1. The length of the box indicates the magnitude of the standard error with respect to the mean. The length of the whiskers indicates the length of the 95% confidence interval

The same patterns can be observed in Figs. 1, 2, 3, 4 and 5, which compare the simulated against estimated standard errors and confidence intervals for a combination of three heritabilities (low, medium, and high) and three proportions of animals with phenotypes (low, medium, and high). For the bias (Fig. 1), the estimation of the confidence intervals was less accurate with low $prop$. Within each $prop$, the approximated and bootstrap standard errors and confidence intervals tend to overestimate the simulated ones as the heritability increases.

The situation was the opposite for the dispersion parameter (Fig. 2). In this case, the approximated and bootstrap confidence intervals were too narrow for high $prop$ with respect to the true confidence intervals obtained from the simulated data. These results suggest that bootstrapping does not consider properly the complex covariance structure between ${\widehat{\mathbf{u}}}_{p}$ and ${\widehat{\mathbf{u}}}_{w}$.

The confidence intervals for the ratio of accuracies were slightly underestimated for the bootstrap method.

The same was observed for the predictivity. However, the variance among replicates was very high for scenarios with low $prop$ or low ${h}^{2}$. In such cases, the confidence intervals for the predictivity would cover a large portion of its range, making inference based on the predictivity statistic inaccurate.

For reliability (Fig. 5), the analytical confidence intervals were very close to the simulated ones. The approximated analytical and the bootstrap confidence intervals were systematically narrower than the simulated ones. In addition, the simulated confidence intervals were not symmetric around the mean, as the lower bound was closer to the mean than the upper bound. This could indicate that approximate normality is not appropriate.

**Fig. 6:**
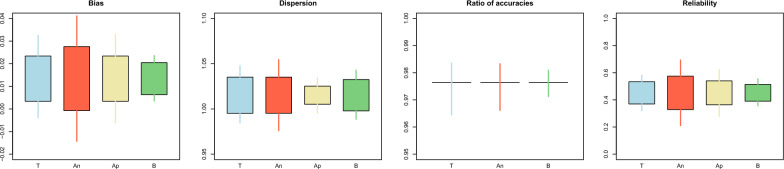
Comparison between the true (T), analytical (An), approximated (Ap), and bootstrap (B) standard error and confidence interval for the estimator of the bias, dispersion, ratio of accuracies^1^, and reliability for the bulls in Example 2. The length of the box indicates the magnitude of the standard error with respect to the mean. The length of the whiskers indicates the length of the 95% confidence interval. ^1^Standard errors were not available for the analytical confidence interval

**Fig. 7:**
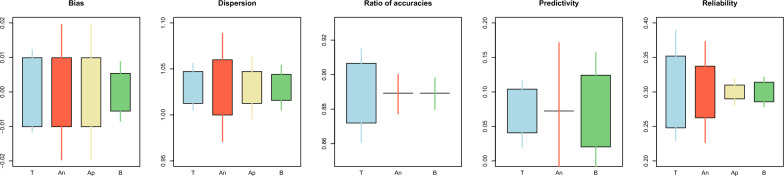
Comparison between the true (T), analytical (An), approximated (Ap), and bootstrap (B) standard error and confidence interval for the estimator of the bias, dispersion, ratio of accuracies^1^, predictivity^1^, and reliability for the cows in Example 2. The length of the box indicates the magnitude of the standard error with respect to the mean. The length of the whiskers indicates the length of the 95% confidence interval. ^1^Standard errors were not available for the analytical confidence interval

Results for Example 2 are shown in Fig. 6 for bulls and Fig. 7 for cows. The analytical confidence intervals for reliabilities in cows were closer to the simulated ones than those for bulls. For bulls, the results were overall more variable and showed that the analytical confidence intervals for all the statistics were biased, probably because bulls were highly selected. This violates the assumption of absence of selection and can affect the expressions involving $\mathbf{G}$. In addition, this issue could have been generated by the sparse inversion [41, 42] implemented in BLUPF90 + , which calculates in an exact manner the elements of ${\mathbf{C}}_{w}^{22}$ and ${\mathbf{C}}_{p}^{22}$ corresponding to the non-zero pattern of the MME and ignores the rest of the elements, which have their values set to zero before sparse inversion. However, these elements, which are not needed for reliabilities or restricted maximum likelihood (REML), are needed to obtain confidence intervals analytically, e.g., in [15]. For instance, the prediction error covariance of two unrelated bulls with daughters in the same herd. Another reason could be that the amount of information removed for the validation bulls was not sufficient, which is shown by a high ${\rho }_{wp}$. Under high ${\rho }_{wp}$, the gain in accuracy from the partial to the whole dataset will be minimal to null, and the standard errors of the validation statistics will tend to zero because ${\mathbf{C}}_{p}^{22}\approx {\mathbf{C}}_{w}^{22}$. Similar to the results from Example 1, the bootstrap confidence intervals were narrower than the simulated ones.

## Discussion

The aim of this study was to derive standard errors and analytical confidence intervals for the LR method and predictivity. For the estimators of the bias, dispersion, and reliability from the LR method, we calculated their standard errors and built Wald confidence intervals assuming that the estimators are asymptotically normally distributed. Unlike [24], we used the marginal (unconditional) distribution of the estimators to account for the randomness of ${\widehat{\mathbf{u}}}_{p}$ and the dependence between ${\widehat{\mathbf{u}}}_{p}$ and ${\widehat{\mathbf{u}}}_{w}$. Not accounting for the randomness of ${\widehat{\mathbf{u}}}_{p}$ results in an underestimation of the standard errors of the validation statistics; hence, resulting in narrower confidence intervals. The resulting standard errors and confidence intervals are functions of the relationships between the individuals in the validation set and their prediction error (co)variances in the whole and partial datasets.

For the estimator of the ratio of accuracies from the LR method and the predictivity, we used the Fisher transformation to obtain a confidence interval of those correlation coefficients. Although this method is straightforward, it assumes that all the samples are identically and independently distributed, which is not true when performing validation by data truncation in genetic evaluations. Looking for better formulas that account for heterogeneity in variances and dependence among samples will involve complicated expressions (see Appendix II). In addition, Krishnamoorthy and Xia [43] and Gnambs [44] showed that Fisher’s transformation worked well with a large number of observations regardless of whether its assumptions were violated. Also, unlike the standard errors for bias, dispersion, and reliability, which depend only on the model [see Eqs. (5), (14), and (24)], the variances for the ratio of accuracies and predictivity depend directly on the values of the statistics themselves.

Although confidence intervals for predictivity can be obtained with Fisher’s transformation, comparing different models based on those confidence intervals is improper because it does not consider the dependency between the statistics. A bootstrap method to account for this was proposed by [24], but parametric methods exist. In other words, the methods presented in this study explain how to obtain confidence intervals for ${\rho }_{{\mathbf{y}}^{*},{\widehat{\mathbf{u}}}_{p}}$ but they do not assess the null (H0) hypothesis ${\rho }_{{\mathbf{y}}^{*},{\widehat{\mathbf{u}}}_{p}(A)}={\rho }_{{\mathbf{y}}^{*},{\widehat{\mathbf{u}}}_{p}(B)}$, where $A$ and $B$ denote different methods or models for prediction. A proper test in this situation is the Williams test [45], which uses the statistic $T=\left({\rho }_{{\mathbf{y}}^{*},{\widehat{\mathbf{u}}}_{p}(A)}-{\rho }_{{\mathbf{y}}^{*},{\widehat{\mathbf{u}}}_{p}(B)}\right)\sqrt{\frac{\left(n-1\right)\left(1+{\rho }_{{\widehat{\mathbf{u}}}_{p}(A),{\widehat{\mathbf{u}}}_{p}(B)}\right)}{2\left(\frac{n-1}{n-3}\right)\left|\mathbf{R}\right|+{\left(\frac{{\rho }_{{\mathbf{y}}^{*},{\widehat{\mathbf{u}}}_{p}(A)}+{\rho }_{{\mathbf{y}}^{*},{\widehat{\mathbf{u}}}_{p}(B)}}{2}\right)}^{2}{\left(\left(1-{\rho }_{{\widehat{\mathbf{u}}}_{p}(A),{\widehat{\mathbf{u}}}_{p}(B)}\right)\right)}^{3}}}$ to compare correlations ${\rho }_{{\mathbf{y}}^{*},{\widehat{\mathbf{u}}}_{p}(A)}$ and ${\rho }_{{\mathbf{y}}^{*},{\widehat{\mathbf{u}}}_{p}(B)}$, where ${\rho }_{{\widehat{\mathbf{u}}}_{p}(A),{\widehat{\mathbf{u}}}_{p}(B)}$ is the correlation between EBV calculated with methods $A$ and $B$, and $\left|\mathbf{R}\right|=\left(1-{\rho }_{{\mathbf{y}}^{*},{\widehat{\mathbf{u}}}_{p}\left(A\right)}-{\rho }_{{\mathbf{y}}^{*},{\widehat{\mathbf{u}}}_{p}\left(B\right)}-{\rho }_{{\widehat{\mathbf{u}}}_{p}(A),{\widehat{\mathbf{u}}}_{p}(B)}\right)+2\left({\rho }_{{\mathbf{y}}^{*},{\widehat{\mathbf{u}}}_{p}(A)}{\rho }_{{\mathbf{y}}^{*},{\widehat{\mathbf{u}}}_{p}(B)}{\rho }_{{\widehat{\mathbf{u}}}_{p}(A),{\widehat{\mathbf{u}}}_{p}(B)}\right)$. Statistic $T$ follows approximately a $t$ distribution with $n-3$ degrees of freedom. Indeed, this test has already been used but not in the context of the LR method [46].

According to the results of our study, analytical confidence intervals should be preferred over bootstrap confidence intervals. However, the analytical confidence intervals for the bias, dispersion, and reliability are computationally expensive to obtain in large datasets because they need the prediction error variances and covariances for the validation animals in the whole and partial datasets. Alternatives for large-scale genetic evaluations could be to approximate ${\mathbf{C}}_{w}^{22}$ and ${\mathbf{C}}_{p}^{22}$ with Markov chain Monte Carlo methods [47]. In this study, the approximations that we propose assume $\left({\mathbf{C}}_{p}^{22}-{\mathbf{C}}_{w}^{22}\right)$ and $\left(\mathbf{G}-{\mathbf{C}}_{p}^{22}\right)$ to be diagonal. In such a case, ${\mathbf{C}}_{w}^{22}$ and ${\mathbf{C}}_{p}^{22}$ can be obtained from the (G)EBV reliabilities reported in the evaluation, which corresponds to, for instance, the information that is used in Interbulls’ tests [48, 49]. The robustness of the diagonal assumption depends on the data. For not-very-related individuals with high reliabilities, the assumption holds. More complex scenarios, for instance, families with half-sibs and low to medium reliabilities will require further inspections due to a block structure of $\left({\mathbf{C}}_{p}^{22}-{\mathbf{C}}_{w}^{22}\right)$ and $\left(\mathbf{G}-{\mathbf{C}}_{p}^{22}\right)$. Assuming that the increase in reliability from partial to whole data $\left(c\right)$ is constant among animals leads to an expression where only $re{l}_{p}$ or $re{l}_{w}$ are required. This could be attractive in cases where performing validation by data truncation is not possible (e.g., when phenotypes could not be shared or $re{l}_{p}$ might not be available) or when adding a source of information or calculating the reliability is not possible ($re{l}_{w}$ might not be available). The assumption of a constant increase in reliability, using the average increase of the reliability for the calculations, was shown to be robust in this study in spite of the range of $c$, which ranged up to (1.86-8.91) for some scenarios in Example 1. In our simulations, the approximated analytical confidence intervals were similar to those obtained by bootstrapping.

In many scenarios in both examples, the bootstrap confidence intervals were narrower than the simulated ones. In other words, bootstrapping was “too optimistic” regardless of the variation of the empirical distribution of the validation statistic. The reason could be the correlated data structure shown by populations under artificial selection. Bickel et al. [50] reviewed situations and presented scenarios where classical bootstrap fails. In such a case, they proposed sampling with replacement using fewer observations than the total number. In addition, this could increase the efficiency of bootstrapping. The number of observations to sample would depend on the data and could be calibrated with the analytical confidence intervals in case they are too expensive to obtain for routine evaluations.

The additive genetic variance and the accuracy of the EBV change when selection occurs [51, 52]. To our knowledge, the interaction between predictivity and selection has not been studied. That statistic depends on the square root of the heritability. Thus, if the estimate of the heritability under selection is biased, the predictivity could also be biased. In case the model and genetic parameters are correct, the predictivity could be biased if selected animals are chosen for the validation set. Simulation studies reported that the LR method worked well when the model used to estimate breeding values matches the true data-generating process [14, 29]. According to these results, one could infer that the LR method would estimate the bias, dispersion, and accuracy properly in the presence of selection if it is correctly taken into account in the model with, for instance, the method of Henderson [53, 54]. However, this is rarely used in genetic evaluations, and selection is often ignored in the estimation of breeding values. In such a case, the LR method can estimate the direction of the bias but not its magnitude if the model is incorrect but reasonably robust [29]. The LR method cannot estimate the bias when the model is seriously mis-specified, which in the case of [29] was when a simulated environmental trend was ignored in the model. Macedo et al. [29] found that the dispersion and accuracy were well estimated in all scenarios. However, Himmelbauer et al. [14] found that the LR method performed well for males but not for females in dairy cattle selection schemes. In addition, they reported that the estimator of the reliability depends heavily on how the additive genetic variance for the validation set is calculated. The confidence intervals derived in this study can be affected by selection in two ways: (i) by the bias of the validation statistics and (ii) by the effect of selection on the standard error of the dispersion and reliability estimators. The first way affects the location of the confidence interval, and given a biased estimator, it is not possible to correct. The second way affects the confidence interval length because the additive relationships in the validation group change due to selection [53, 54]. Specifically, the affected term is $\left(\mathbf{G}-{\mathbf{C}}_{p}^{22}\right)$, which is the variance of ${\widehat{\mathbf{u}}}_{p}$. According to Henderson [53, 54], the variance of ${\widehat{\mathbf{u}}}_{p}$ is reduced under selection. However, the effect on the standard error of the estimators of the dispersion and reliability is hard to assess because the variance of ${\widehat{\mathbf{u}}}_{p}$ is involved in convoluted algebraic operations.

## Conclusions

We derived analytical standard errors and confidence intervals for predictivity and the LR method statistics of bias, dispersion, ratio of accuracies, and reliability. Based on the examples shown in this study, the analytical confidence intervals were more accurate than the confidence intervals obtained by bootstrapping. We also developed approximated analytical confidence intervals for situations where the analytical ones are not feasible due to computational limitations. This study provided a framework for proper validation by data truncation statistical inference applied to genetic evaluation when replication is not possible.

### Supplementary Information

**Additional file 1: Table S1.** Average squared differences between estimated and true variance, lower bound of the 95% confidence interval (lCI), and upper bound of the 95% confidence interval (uCI) for Example 1 for different heritabilities. **Table S2.** Average squared differences between estimated and true variance, lower bound of the 95% confidence interval (lCI), and upper bound of the 95% confidence interval (uCI) for Example 1 for different proportions of phenotyped animals.

## Data Availability

Data and material are available upon request.

## Appendix I

This is essentially a rewriting of properties shown by several authors [53–56], some of them difficult to find, that we put together here for readers’ convenience. The full model is:

29 $$\mathbf{y}=\mathbf{X}\mathbf{b}+\mathbf{Z}\mathbf{u}+\mathbf{e}.$$

Assuming $\mathbf{u}\sim {\text{MVN}}({\mathbf{0}},\mathbf{G})$, $\mathbf{e}\sim {\text{MVN}}({\mathbf{0}},\mathbf{R})$, and ${\text{cov}}\left(\mathbf{u},\mathbf{e}\right)=0$ gives $\mathbf{y}\sim {\text{MVN}}(\mathbf{X}\mathbf{b},\mathbf{V}=\mathbf{Z}\mathbf{G}{\mathbf{Z}}^{\prime}+\mathbf{R})$. The best linear unbiased prediction of $\mathbf{u}$ is obtained as ${\widehat{\mathbf{u}}}_{w}={\mathbf{C}}_{w}\mathbf{y}=\left(\mathbf{G}{\mathbf{Z}}^{\prime}{\mathbf{V}}^{-1}-\mathbf{G}{\mathbf{Z}}^{\prime}{\mathbf{V}}^{-1}\mathbf{X}{\left({\mathbf{X}}^{\prime}{\mathbf{V}}^{-1}\mathbf{X}\right)}^{-}{\mathbf{X}}^{\prime}{\mathbf{V}}^{-1}\right)\mathbf{y}$. The partial model (the model without the phenotypes of the validation set) is:

30 $${\mathbf{y}}_{p}={\mathbf{X}}_{p}{\mathbf{b}}_{p}+{\mathbf{Z}}_{p}\mathbf{u}+{\mathbf{e}}_{p}.$$

In this case, the best linear unbiased prediction of $\mathbf{u}$ is obtained as $${\widehat{\mathbf{u}}}_{p}={\mathbf{C}}_{p}\mathbf{y}=\left[\begin{array}{cc}\left(\mathbf{G}{\mathbf{Z}}_{p}^{\prime}{\mathbf{V}}_{p}^{-1}-\mathbf{G}{\mathbf{Z}}_{p}^{\prime}{\mathbf{V}}_{p}^{-1}{\mathbf{X}}_{p}{\left({\mathbf{X}}_{p}^{\prime}{\mathbf{V}}_{p}^{-1}{\mathbf{X}}_{p}\right)}^{-}{\mathbf{X}}_{p}^{\prime}{\mathbf{V}}_{p}^{-1}\right)& {\mathbf{0}}\end{array}\right]\left[\begin{array}{c}{\mathbf{y}}_{p}\\ {\mathbf{y}}_{w-{\varvec{p}}}\end{array}\right]$$. Then:

31 $$\left[\begin{array}{c}{\widehat{\mathbf{u}}}_{w}\\ {\widehat{\mathbf{u}}}_{p}\end{array}\right]=\left[\begin{array}{c}{\mathbf{C}}_{w}\\ {\mathbf{C}}_{p}\end{array}\right]\mathbf{y}.$$

By affine transformation of $\mathbf{y}$ (see p. 92 in [25]):

32 $$\left[\begin{array}{c}{\widehat{\mathbf{u}}}_{w}\\ {\widehat{\mathbf{u}}}_{p}\end{array}\right]\sim {\text{MVN}}\left(\left[\begin{array}{c}{\text{E}}\left[{\mathbf{C}}_{w}\mathbf{y}\right]\\ {\text{E}}\left[{\mathbf{C}}_{p}\mathbf{y}\right]\end{array}\right],\left[\begin{array}{cc}{\mathbf{C}}_{w}\mathbf{V}{\mathbf{C}}_{w}^{\mathrm{^{\prime}}}& {\mathbf{C}}_{w}\mathbf{V}{\mathbf{C}}_{p}^{\mathrm{^{\prime}}}\\ {\mathbf{C}}_{p}\mathbf{V}{\mathbf{C}}_{w}^{\mathrm{^{\prime}}}& {\mathbf{C}}_{p}\mathbf{V}{\mathbf{C}}_{p}^{\mathrm{^{\prime}}}\end{array}\right]\right),$$

which results in [3, 55]:

33 $$\left[\begin{array}{c}{\widehat{\mathbf{u}}}_{w}\\ {\widehat{\mathbf{u}}}_{p}\end{array}\right]\sim {\text{MVN}}\left(\left[\begin{array}{c}{\mathbf{0}}\\ {\mathbf{0}}\end{array}\right],\left[\begin{array}{cc}\mathbf{G}-{\mathbf{C}}_{w}^{22}& \mathbf{G}-{\mathbf{C}}_{p}^{22}\\ \mathbf{G}-{\mathbf{C}}_{p}^{22}& \mathbf{G}-{\mathbf{C}}_{p}^{22}\end{array}\right]\right).$$

To account for selection, [29] showed that $\mathbf{G}$ should be replaced by ${\mathbf{G}}^{*}=\mathbf{G}-{\mathbf{B}}_{u}{\mathbf{H}}_{0}{\mathbf{B}}_{u}^{\prime}$ [53], where ${\mathbf{B}}_{u}$ represents the selection process and ${\mathbf{H}}_{0}$ represents the decrease in variance under selection. However, information about the selection process to write down matrices ${\mathbf{B}}_{u}$ and ${\mathbf{H}}_{0}$ is unknown, except for idealized settings. For this reason and for simplicity, absence of selection is assumed to result in Eq. (33). Then, by multivariate normality, ${\widehat{\mathbf{u}}}_{w}|{\widehat{\mathbf{u}}}_{p}$ follows a multivariate normality distribution with mean:

34 $${\text{E}}\left[{\widehat{\mathbf{u}}}_{w}|{\widehat{\mathbf{u}}}_{p}\right]={\text{Cov}}\left({\widehat{\mathbf{u}}}_{w},{\widehat{\mathbf{u}}}_{p}\right){\text{Var}}{\left({\widehat{\mathbf{u}}}_{p}\right)}^{-1}\left({\widehat{\mathbf{u}}}_{p}-{\text{E}}\left[{\widehat{\mathbf{u}}}_{w}\right]\right)=\left(\mathbf{G}-{\mathbf{C}}_{p}^{22}\right){\left(\mathbf{G}-{\mathbf{C}}_{p}^{22}\right)}^{-1}{\widehat{\mathbf{u}}}_{p}={\widehat{\mathbf{u}}}_{p},$$

and variance:

35 $${\text{Var}}\left({\widehat{\mathbf{u}}}_{w}|{\widehat{\mathbf{u}}}_{p}\right)={\text{Var}}\left({\widehat{\mathbf{u}}}_{w}\right)-{\text{Cov}}\left({\widehat{\mathbf{u}}}_{w},{\widehat{\mathbf{u}}}_{p}\right){\text{Var}}{\left({\widehat{\mathbf{u}}}_{p}\right)}^{-1}{\text{Cov}}\left({\widehat{\mathbf{u}}}_{p},{\widehat{\mathbf{u}}}_{w}\right)=\left(\mathbf{G}-{\mathbf{C}}_{w}^{22}\right)-\left(\mathbf{G}-{\mathbf{C}}_{p}^{22}\right){\left(\mathbf{G}-{\mathbf{C}}_{p}^{22}\right)}^{-1}\left(\mathbf{G}-{\mathbf{C}}_{p}^{22}\right)={\mathbf{C}}_{p}^{22}-{\mathbf{C}}_{w}^{22}.$$

Thus,

36 $${\widehat{\mathbf{u}}}_{w}|{\widehat{\mathbf{u}}}_{p}\sim {\text{MVN}}\left({\widehat{\mathbf{u}}}_{p},{\mathbf{C}}_{p}^{22}-{\mathbf{C}}_{w}^{22}\right).$$

## Appendix II

Let $r=\frac{{\sigma }_{12}}{\sqrt{{\sigma }_{1}^{2}{\sigma }_{2}^{2}}}$ be a Pearson sample correlation coefficient. Using a first-order Taylor approximation, its variance is:

37 $${\text{Var}}\left(r\right)={r}^{2}\left(\frac{{\text{Var}}\left({\sigma }_{1}^{2}\right)}{4{\sigma }_{1}^{4}}+\frac{{\text{Var}}\left({\sigma }_{2}^{2}\right)}{4{\sigma }_{2}^{4}}+\frac{{\text{Var}}\left({\sigma }_{12}\right)}{{\sigma }_{12}^{2}}+\frac{{\text{Cov}}\left({\sigma }_{1}^{2},{\sigma }_{2}^{2}\right)}{2{\sigma }_{1}^{2}{\sigma }_{2}^{2}}-\frac{{\text{Cov}}\left({\sigma }_{1}^{2},{\sigma }_{12}\right)}{{\sigma }_{1}^{2}{\sigma }_{12}}-\frac{{\text{Cov}}\left({\sigma }_{12},{\sigma }_{2}^{2}\right)}{{\sigma }_{12}{\sigma }_{2}^{2}}\right).$$

Applying Eq. (37) to the estimator of the ratio of accuracies $\left({\rho }_{wp}\right)$ (Eq. 18) we have:

38 $$\begin{aligned}{\text{Var}}\left(\frac{{\widehat{\mathbf{u}}}_{w}^{\prime}\mathbf{S}\,{\widehat{\mathbf{u}}}_{p}}{\sqrt{{\widehat{{\mathbf{u}}}_{p}{^{\prime}}}\mathbf{S}\,{\widehat{\mathbf{u}}}_{p} {\widehat{\mathbf{u}}}_{w}^{\prime}\mathbf{S}\,{\widehat{\mathbf{u}}}_{w}}}\right)={\left(\frac{{\widehat{\mathbf{u}}}_{w}^{\prime}\mathbf{S}\,{\widehat{\mathbf{u}}}_{p}}{\sqrt{{\widehat{\mathbf{u}}}_{p}^{\prime}\mathbf{S}\,{\widehat{\mathbf{u}}}_{p} {\widehat{\mathbf{u}}}_{w}^{\prime}\mathbf{S}\,{\widehat{\mathbf{u}}}_{w}}}\right)}^{2}\left(\frac{{\text{Var}}\left({\widehat{\mathbf{u}}}_{p}^{\prime}\mathbf{S}\,{\widehat{\mathbf{u}}}_{p}\right)}{4{\left({\widehat{\mathbf{u}}}_{p}^{\prime}\mathbf{S}\,{\widehat{\mathbf{u}}}_{p}\right)}^{2}}  + \frac{{\text{Var}}\left( {\widehat{\mathbf{u}}}_{w}^{\prime}\mathbf{S}\,{\widehat{\mathbf{u}}}_{w}\right)}{4{\left( {\widehat{\mathbf{u}}}_{w}^{\prime}\mathbf{S}\,{\widehat{\mathbf{u}}}_{w}\right)}^{2}}+\frac{{\text{Var}}\left({\widehat{\mathbf{u}}}_{w}^{\prime}\mathbf{S}\,{\widehat{\mathbf{u}}}_{p}\right)}{{\left({\widehat{\mathbf{u}}}_{w}^{\prime}\mathbf{S}\,{\widehat{\mathbf{u}}}_{p}\right)}^{2}} +\frac{{\text{Cov}}\left({\widehat{\mathbf{u}}}_{p}^{\prime}\mathbf{S}\,{\widehat{\mathbf{u}}}_{p}, {\widehat{\mathbf{u}}}_{w}^{\prime}\mathbf{S}\,{\widehat{\mathbf{u}}}_{w}\right)}{2 {\widehat{\mathbf{u}}}_{p}^{\prime}\mathbf{S}\,{\widehat{\mathbf{u}}}_{p} {\widehat{\mathbf{u}}}_{w}^{\prime}\mathbf{S}\,{\widehat{\mathbf{u}}}_{w}}-\frac{{\text{Cov}}\left({\widehat{\mathbf{u}}}_{p}^{\prime}\mathbf{S}\,{\widehat{\mathbf{u}}}_{p},{\widehat{\mathbf{u}}}_{w}^{\prime}\mathbf{S}\,{\widehat{\mathbf{u}}}_{p}\right)}{{\widehat{\mathbf{u}}}_{p}^{\prime}\mathbf{S}\,{\widehat{\mathbf{u}}}_{p} {\widehat{\mathbf{u}}}_{w}^{\prime}\mathbf{S}\,{\widehat{\mathbf{u}}}_{p}}-\frac{{\text{Cov}}\left({\widehat{\mathbf{u}}}_{w}^{\prime}\mathbf{S}\,{\widehat{\mathbf{u}}}_{p}, {\widehat{\mathbf{u}}}_{w}^{\prime}\mathbf{S}\,{\widehat{\mathbf{u}}}_{w}\right)}{{\widehat{\mathbf{u}}}_{w}^{\prime}\mathbf{S}\,{\widehat{\mathbf{u}}}_{p} {\widehat{\mathbf{u}}}_{w}^{\prime}\mathbf{S}\,{\widehat{\mathbf{u}}}_{w}}\right). \end{aligned}$$

Recalling that ${\text{Var}}\left({\widehat{\mathbf{u}}}_{p}^{\prime}\mathbf{S}{\widehat{\mathbf{u}}}_{p}\right)=2\,{\text{tr}}\left(\mathbf{S}\left(\mathbf{G}-{\mathbf{C}}_{p}^{22}\right)\mathbf{S}\left(\mathbf{G}-{\mathbf{C}}_{p}^{22}\right)\right)$, ${\text{Var}}\left({\widehat{\mathbf{u}}}_{w}^{\prime}\mathbf{S}{\widehat{\mathbf{u}}}_{w}\right)=2\,{\text{tr}}\left(\mathbf{S}\left(\mathbf{G}-{\mathbf{C}}_{w}^{22}\right)\mathbf{S}\left(\mathbf{G}-{\mathbf{C}}_{w}^{22}\right)\right)$, and ${\text{Var}}\left({\widehat{\mathbf{u}}}_{p}^{\prime}\mathbf{S}{\widehat{\mathbf{u}}}_{w}\right)={\text{tr}}\left(\mathbf{S}\left({\mathbf{C}}_{p}^{22}-{\mathbf{C}}_{w}^{22}\right)\mathbf{S}\left(\mathbf{G}-{\mathbf{C}}_{p}^{22}\right)\right)+2\,{\text{tr}}\left(\mathbf{S}\left(\mathbf{G}-{\mathbf{C}}_{p}^{22}\right)\mathbf{S}\left(\mathbf{G}-{\mathbf{C}}_{p}^{22}\right)\right)$**,** it can proved using (see p. 66 [32]) that ${\text{Cov}}\left({\widehat{\mathbf{u}}}_{p}^{\prime}\mathbf{S}{\widehat{\mathbf{u}}}_{p}, {\widehat{\mathbf{u}}}_{w}^{\prime}\mathbf{S}{\widehat{\mathbf{u}}}_{w}\right)={\text{Cov}}\left({\widehat{\mathbf{u}}}_{p}^{\prime}\mathbf{S}{\widehat{\mathbf{u}}}_{p}, {\widehat{\mathbf{u}}}_{p}^{\prime}\mathbf{S}{\widehat{\mathbf{u}}}_{w}\right)={\text{Var}}\left({\widehat{\mathbf{u}}}_{p}^{\prime}\mathbf{S}{\widehat{\mathbf{u}}}_{p}\right)$ and ${\text{Cov}}\left({\widehat{\mathbf{u}}}_{w}^{\prime}\mathbf{S}{\widehat{\mathbf{u}}}_{p}, {\widehat{\mathbf{u}}}_{w}^{\prime}\mathbf{S}{\widehat{\mathbf{u}}}_{w}\right)=2\,{\text{tr}}\left(\mathbf{S}\left(\mathbf{G}-{\mathbf{C}}_{w}^{22}\right)\mathbf{S}\left(\mathbf{G}-{\mathbf{C}}_{p}^{22}\right)\right)$. Then, Eq. (38) results in:

39 $${\text{Var}}\left(\frac{{\widehat{\mathbf{u}}}_{w}^{\prime}\mathbf{S}\,{\widehat{\mathbf{u}}}_{p}}{\sqrt{{\widehat{\mathbf{u}}}_{p}^{\prime}\mathbf{S}\,{\widehat{\mathbf{u}}}_{p} {\widehat{\mathbf{u}}}_{w}^{\prime}\mathbf{S}\,{\widehat{\mathbf{u}}}_{w}}}\right)={\left(\frac{{\widehat{\mathbf{u}}}_{w}^{\prime}\mathbf{S}\,{\widehat{\mathbf{u}}}_{p}}{\sqrt{{\widehat{\mathbf{u}}}_{p}^{\prime}\mathbf{S}\,{\widehat{\mathbf{u}}}_{p} {\widehat{\mathbf{u}}}_{w}^{\prime}\mathbf{S}\,{\widehat{\mathbf{u}}}_{w}}}\right)}^{2}\left(\frac{2\,{\text{tr}}\left(\mathbf{S}\left(\mathbf{G}-{\mathbf{C}}_{p}^{22}\right)\mathbf{S}\left(\mathbf{G}-{\mathbf{C}}_{p}^{22}\right)\right)}{4{\left({\widehat{\mathbf{u}}}_{p}^{\prime}\mathbf{S}\,{\widehat{\mathbf{u}}}_{p}\right)}^{2}}+\frac{2\,{\text{tr}}\left(\mathbf{S}\left(\mathbf{G}-{\mathbf{C}}_{w}^{22}\right)\mathbf{S}\left(\mathbf{G}-{\mathbf{C}}_{w}^{22}\right)\right)}{4{\left( {\widehat{\mathbf{u}}}_{w}^{\prime}\mathbf{S}\,{\widehat{\mathbf{u}}}_{w}\right)}^{2}}+\frac{{\text{tr}}\left(\mathbf{S}\left({\mathbf{C}}_{p}^{22}-{\mathbf{C}}_{w}^{22}\right)\mathbf{S}\left(\mathbf{G}-{\mathbf{C}}_{p}^{22}\right)\right)+2\,{\text{tr}}\left(\mathbf{S}\left(\mathbf{G}-{\mathbf{C}}_{p}^{22}\right)\mathbf{S}\left(\mathbf{G}-{\mathbf{C}}_{p}^{22}\right)\right)}{{\left({\widehat{\mathbf{u}}}_{w}^{\prime}\mathbf{S}\,{\widehat{\mathbf{u}}}_{p}\right)}^{2}}+\frac{2\,{\text{tr}}\left(\mathbf{S}\left(\mathbf{G}-{\mathbf{C}}_{p}^{22}\right)\mathbf{S}\left(\mathbf{G}-{\mathbf{C}}_{p}^{22}\right)\right)}{2 {\widehat{\mathbf{u}}}_{p}^{\prime}\mathbf{S}\,{\widehat{\mathbf{u}}}_{p} {\widehat{\mathbf{u}}}_{w}^{\prime}\mathbf{S}\,{\widehat{\mathbf{u}}}_{w}}-\frac{2\,{\text{tr}}\left(\mathbf{S}\left(\mathbf{G}-{\mathbf{C}}_{p}^{22}\right)\mathbf{S}\left(\mathbf{G}-{\mathbf{C}}_{p}^{22}\right)\right)}{{\widehat{\mathbf{u}}}_{p}^{\prime}\mathbf{S}\,{\widehat{\mathbf{u}}}_{p} {\widehat{\mathbf{u}}}_{w}^{\prime}\mathbf{S}{\widehat{\mathbf{u}}}_{p}}-\frac{2\,{\text{tr}}\left(\mathbf{S}\left(\mathbf{G}-{\mathbf{C}}_{w}^{22}\right)\mathbf{S}\left(\mathbf{G}-{\mathbf{C}}_{p}^{22}\right)\right)}{{\widehat{\mathbf{u}}}_{w}^{\prime}\mathbf{S}{\widehat{\mathbf{u}}}_{p} {\widehat{\mathbf{u}}}_{w}^{\prime}\mathbf{S}\,{\widehat{\mathbf{u}}}_{w}}\right).$$

Then, applying Eq. (37) to the predictive ability ${\rho }_{{\mathbf{y}}^{*},{\widehat{\mathbf{u}}}_{p}}=\frac{1}{h}\frac{cov\left({\mathbf{y}}^{*},{\widehat{\mathbf{u}}}_{p}\right)}{\sqrt{var\left({\mathbf{y}}^{*}\right) var\left({\widehat{\mathbf{u}}}_{p}\right)}}$ (Eq. 27) we have:

40 $${\text{Var}}\left(\frac{1}{h}\frac{{{\mathbf{y}}^{\mathbf{*}}}^{\mathbf{^{\prime}}}\mathbf{S}\,{\widehat{\mathbf{u}}}_{p}}{\sqrt{{\widehat{\mathbf{u}}}_{p}{\prime}\mathbf{S}\,{\widehat{\mathbf{u}}}_{p}\, {{\mathbf{y}}^{\mathbf{*}}}^{\mathbf{^{\prime}}}\mathbf{S}\,{\mathbf{y}}^{*}}}\right)=\frac{1}{{h}^{2}}{\left(\frac{{{\mathbf{y}}^{\mathbf{*}}}^{\mathbf{^{\prime}}}\mathbf{S}\,{\widehat{\mathbf{u}}}_{p}}{\sqrt{{\widehat{\mathbf{u}}}_{p}^{\prime}\mathbf{S}\,{\widehat{\mathbf{u}}}_{p}\,{{\mathbf{y}}^{\mathbf{*}}}^{\mathbf{^{\prime}}}\mathbf{S}\,{\mathbf{y}}^{*}}}\right)}^{2}\left(\frac{{\text{Var}}\left({\widehat{\mathbf{u}}}_{p}^{\prime}\mathbf{S}\,{\widehat{\mathbf{u}}}_{p}\right)}{4{\left({\widehat{\mathbf{u}}}_{p}^{\prime}\,\mathbf{S}\,{\widehat{\mathbf{u}}}_{p}\right)}^{2}}+\frac{{\text{Var}}\left({{\mathbf{y}}^{\mathbf{*}}}^{\mathbf{^{\prime}}}\mathbf{S}{\mathbf{y}}^{*}\right)}{4{\left({{\mathbf{y}}^{\mathbf{*}}}^{\mathbf{^{\prime}}}\,\mathbf{S}{\mathbf{y}}^{*}\right)}^{2}}+\frac{{\text{Var}}\left({{\mathbf{y}}^{\mathbf{*}}}^{\mathbf{^{\prime}}}\mathbf{S}\,{\widehat{\mathbf{u}}}_{p}\right)}{{\left({{\mathbf{y}}^{\mathbf{*}}}^{\mathbf{^{\prime}}}\,\mathbf{S}\,{\widehat{\mathbf{u}}}_{p}\right)}^{2}}+\frac{{\text{Cov}}\left({{\widehat{\mathbf{u}}}_{p}^{\prime}}\,\mathbf{S}\,{\widehat{\mathbf{u}}}_{p}\, {{\mathbf{y}}^{\mathbf{*}}}^{\mathbf{^{\prime}}}\mathbf{S}\,{\mathbf{y}}^{*}\right)}{2 {\widehat{\mathbf{u}}}_{p}^{\prime}\,\mathbf{S}\,{\widehat{\mathbf{u}}}_{p} {{\mathbf{y}}^{\mathbf{*}}}^{\mathbf{^{\prime}}}\mathbf{S}\,{\mathbf{y}}^{*}}-\frac{{\text{Cov}}\left({\widehat{\mathbf{u}}}_{p}^{\prime}\mathbf{S}\,{\widehat{\mathbf{u}}}_{p}\,{{\mathbf{y}}^{\mathbf{*}}}^{\mathbf{^{\prime}}}\mathbf{S}\,{\widehat{\mathbf{u}}}_{p}\right)}{{\widehat{\mathbf{u}}}_{p}^{\prime}\mathbf{S}\,{\widehat{\mathbf{u}}}_{p}\,{{\mathbf{y}}^{\mathbf{*}}}^{\mathbf{^{\prime}}}\,\mathbf{S}\,{\widehat{\mathbf{u}}}_{p}}-\frac{{\text{Cov}}\left({{\mathbf{y}}^{\mathbf{*}}}^{\mathbf{^{\prime}}}\mathbf{S}\,{\widehat{\mathbf{u}}}_{p}\,{{\mathbf{y}}^{\mathbf{*}}}^{\mathbf{^{\prime}}}\mathbf{S}\,{\mathbf{y}}^{*}\right)}{{{\mathbf{y}}^{\mathbf{*}}}^{\mathbf{^{\prime}}}\mathbf{S}\,{\widehat{\mathbf{u}}}_{p}\, {{\mathbf{y}}^{\mathbf{*}}}^{\mathbf{^{\prime}}}\mathbf{S}\,{\mathbf{y}}^{*}}\right).$$

The joint distribution of ${\widehat{\mathbf{u}}}_{p}$ and ${\mathbf{y}}^{*}$ is [3]:

41 $$\left[\begin{array}{c}{\widehat{\mathbf{u}}}_{p}\\ {\mathbf{y}}^{*}\end{array}\right]\sim \left[\begin{array}{cc}\left(\mathbf{G}-{\mathbf{C}}_{p}^{22}\right)& \left(\mathbf{G}-{\mathbf{C}}_{p}^{22}\right)\\ \left(\mathbf{G}-{\mathbf{C}}_{p}^{22}\right)& \mathbf{K}\end{array}\right],$$

where $\mathbf{K}=\mathbf{G}+\mathbf{R}-\mathbf{X}{\mathbf{C}}^{11}\mathbf{X}$, with $\mathbf{R}={\text{Var}}\left(\mathbf{e}\right)$ and ${\mathbf{C}}^{11}$ the block of the generalized inverse of the mixed model equations pertaining to the fixed effects. After algebra, ${\text{Var}}\left({{\mathbf{y}}^{\mathbf{*}}}^{\mathbf{^{\prime}}}\,\mathbf{S}{\mathbf{y}}^{*}\right)=2\,{\text{tr}}\left(\mathbf{S}\mathbf{K}\mathbf{S}\mathbf{K}\right)$, ${\text{Var}}\left({{\mathbf{y}}^{\mathbf{*}}}^{\mathbf{^{\prime}}}\mathbf{S}\,{\widehat{\mathbf{u}}}_{p}\right)={\text{tr}}\left(\mathbf{S}\mathbf{L}\mathbf{S}\left(\mathbf{G}-{\mathbf{C}}_{p}^{22}\right)\right)+{\text{tr}}\left(\mathbf{S}\left(\mathbf{G}-{\mathbf{C}}_{p}^{22}\right)\mathbf{S}\left(\mathbf{G}-{\mathbf{C}}_{p}^{22}\right)\right)$ where $\mathbf{L}={\mathbf{C}}_{p}^{22}+\mathbf{R}-\mathbf{X}{\mathbf{C}}^{11}\mathbf{X}$, ${\text{Cov}}\left({\widehat{\mathbf{u}}}_{p}^{\prime}\mathbf{S}{\widehat{\mathbf{u}}}_{p}, {{\mathbf{y}}^{\mathbf{*}}}^{\mathbf{^{\prime}}}\mathbf{S}{\mathbf{y}}^{*}\right)={\text{Cov}}\left({\widehat{\mathbf{u}}}_{p}^{\prime}\mathbf{S}{\widehat{\mathbf{u}}}_{p},{{\mathbf{y}}^{\mathbf{*}}}^{\mathbf{^{\prime}}}\mathbf{S}{\widehat{\mathbf{u}}}_{p}\right)={\text{Var}}\left({\widehat{\mathbf{u}}}_{p}^{\prime}\mathbf{S}{\widehat{\mathbf{u}}}_{p}\right)$, and ${\text{Cov}}\left({{\mathbf{y}}^{\mathbf{*}}}^{\mathbf{^{\prime}}}\mathbf{S}{\widehat{\mathbf{u}}}_{p},{{\mathbf{y}}^{\mathbf{*}}}^{\mathbf{^{\prime}}}\mathbf{S}{\mathbf{y}}^{*}\right)=2\,{\text{tr}}\left(\mathbf{S}\mathbf{K}\mathbf{S}\left(\mathbf{G}-{\mathbf{C}}_{p}^{22}\right)\right)$. Then, Eq. (37) results in:

42 $${\text{Var}}\left(\frac{1}{h}\frac{{{\mathbf{y}}^{\mathbf{*}}}^{\mathbf{^{\prime}}}\, \mathbf{S}\,{\widehat{\mathbf{u}}}_{p}}{\sqrt{{\widehat{\mathbf{u}}}_{p}^{\prime}\mathbf{S}\,{\widehat{\mathbf{u}}}_{p} {{\mathbf{y}}^{\mathbf{*}}}^{\mathbf{^{\prime}}}\,\mathbf{S}\,{\mathbf{y}}^{*}}}\right)=\frac{1}{{h}^{2}}{\left(\frac{{{\mathbf{y}}^{\mathbf{*}}}^{\mathbf{^{\prime}}}\,\mathbf{S}\,{\widehat{\mathbf{u}}}_{p}}{\sqrt{{\widehat{\mathbf{u}}}_{p}^{\prime}\,\mathbf{S}\,{\widehat{\mathbf{u}}}_{p}\, {{\mathbf{y}}^{\mathbf{*}}}^{\mathbf{^{\prime}}}\, \mathbf{S}\,{\mathbf{y}}^{*}}}\right)}^{2}\left(\frac{2\,{\text{tr}}\left(\mathbf{S}\left(\mathbf{G}-{\mathbf{C}}_{p}^{22}\right)\mathbf{S}\left(\mathbf{G}-{\mathbf{C}}_{p}^{22}\right)\right)}{4{\left({\widehat{\mathbf{u}}}_{p}^{\prime}\,\mathbf{S}\,{\widehat{\mathbf{u}}}_{p}\right)}^{2}}+\frac{2\,{\text{tr}}\left(\mathbf{S}\mathbf{K}\mathbf{S}\mathbf{K}\right)}{4{\left({{\mathbf{y}}^{\mathbf{*}}}^{\mathbf{^{\prime}}}\,\mathbf{S}\,{\mathbf{y}}^{*}\right)}^{2}}+\frac{{\text{tr}}\left(\mathbf{S}\mathbf{L}\mathbf{S}\left(\mathbf{G}-{\mathbf{C}}_{p}^{22}\right)\right)+{\text{tr}}\left(\mathbf{S}\left(\mathbf{G}-{\mathbf{C}}_{p}^{22}\right)\mathbf{S}\left(\mathbf{G}-{\mathbf{C}}_{p}^{22}\right)\right)}{{\left({{\mathbf{y}}^{\mathbf{*}}}^{\mathbf{^{\prime}}}\,\mathbf{S}\, {\widehat{\mathbf{u}}}_{p}\right)}^{2}}+\frac{2\,{\text{tr}}\left(\mathbf{S}\left(\mathbf{G}-{\mathbf{C}}_{p}^{22}\right)\mathbf{S}\left(\mathbf{G}-{\mathbf{C}}_{p}^{22}\right)\right)}{2 {\widehat{\mathbf{u}}}_{p}^{\prime}\,\mathbf{S}\,{\widehat{\mathbf{u}}}_{p}\, {{\mathbf{y}}^{\mathbf{*}}}^{\mathbf{^{\prime}}}\,\mathbf{S}\,{\mathbf{y}}^{*}}-\frac{2\,{\text{tr}}\left(\mathbf{S}\left(\mathbf{G}-{\mathbf{C}}_{p}^{22}\right)\mathbf{S}\left(\mathbf{G}-{\mathbf{C}}_{p}^{22}\right)\right)}{{\widehat{\mathbf{u}}}_{p}^{\prime}\,\mathbf{S}\,{\widehat{\mathbf{u}}}_{p}\, {{\mathbf{y}}^{\mathbf{*}}}^{\mathbf{^{\prime}}}\,\mathbf{S}\,{\widehat{\mathbf{u}}}_{p}}-\frac{2\,{\text{tr}}\left(\mathbf{S}\mathbf{K}\mathbf{S}\left(\mathbf{G}-{\mathbf{C}}_{p}^{22}\right)\right)}{{{\mathbf{y}}^{\mathbf{*}}}^{\mathbf{^{\prime}}}\,\mathbf{S}\,{\widehat{\mathbf{u}}}_{p}\, {{\mathbf{y}}^{\mathbf{*}}}^{\mathbf{^{\prime}}}\,\mathbf{S}\,{\mathbf{y}}^{*}}\right).$$
