# Supplementary material for: Confidence intervals for validation statistics with data truncation in genomic prediction
Source: Genet Sel Evol. 2024 Mar 8;56:18. doi: 10.1186/s12711-024-00883-w (PMC11234739; doi:10.1186/s12711-024-00883-w)
Supplement: Supplementary file 1 — Additional file 1: Table S1. Average squared differences between estimated and true variance, lower bound of the 95% confidence interval (lCI), and upper bound of the 95% confidence interval (uCI) for Example 1 for different heritabilities. Table S2. Average squared differences between estimated and true variance, lower bound of the 95% confidence interval (lCI), and upper bound of the 95% confidence interval (uCI) for Example 1 for different proportions of phenotyped animals. [file 12711_2024_883_MOESM1_ESM.docx]

**Supplementary Table 1.** Average squared differences between estimated and true variance, lower bound of the 95% confidence interval (lCI), and upper bound of the 95% confidence interval (uCI) for Example 1 for different heritabilities.

|  |  |  | Var | lCI | uCI |
| --- | --- | --- | --- | --- | --- |
| h2=0.9 | Bias | Analytical | 1.67E-08 | 8.38E-04 | 1.31E-03 |
|  |  | Approximated | 2.61E-05 | 6.37E-03 | 9.53E-03 |
|  |  | Bootstrap | 3.29E-05 | 4.24E-03 | 6.55E-03 |
|  | Dispersion | Analytical | 3.27E-05 | 4.00E-02 | 8.71E-02 |
|  |  | Approximated | 3.29E-05 | 4.25E-02 | 8.09E-02 |
|  |  | Bootstrap | 1.88E-04 | 6.06E-02 | 8.57E-02 |
|  | Reliability | Analytical | 3.51E-06 | 2.20E-02 | 9.81E-03 |
|  |  | Approximated | 5.01E-05 | 1.04E-02 | 2.36E-02 |
|  |  | Bootstrap | 6.74E-05 | 7.91E-03 | 3.58E-02 |
|  | Ratio of accuracies | Analytical |  | 1.26E-02 | 7.21E-03 |
|  |  | Bootstrap | 1.68E-05 | 1.54E-02 | 7.79E-03 |
|  | Predictivity | Analytical |  | 1.48E-02 | 9.20E-03 |
|  |  | Bootstrap | 2.07E-05 | 1.83E-02 | 9.65E-03 |
| h2=0.8 | Bias | Analytical | 2.78E-08 | 8.38E-04 | 1.24E-03 |
|  |  | Approximated | 2.06E-05 | 5.83E-03 | 8.23E-03 |
|  |  | Bootstrap | 2.66E-05 | 3.99E-03 | 5.74E-03 |
|  | Dispersion | Analytical | 3.24E-05 | 4.11E-02 | 8.31E-02 |
|  |  | Approximated | 4.30E-05 | 4.45E-02 | 7.48E-02 |
|  |  | Bootstrap | 1.82E-04 | 6.31E-02 | 8.16E-02 |
|  | Reliability | Analytical | 3.11E-06 | 2.07E-02 | 9.22E-03 |
|  |  | Approximated | 4.49E-05 | 8.98E-03 | 2.40E-02 |
|  |  | Bootstrap | 5.91E-05 | 6.58E-03 | 3.60E-02 |
|  | Ratio of accuracies | Analytical |  | 1.30E-02 | 7.61E-03 |
|  |  | Bootstrap | 2.00E-05 | 1.57E-02 | 8.47E-03 |
|  | Predictivity | Analytical |  | 1.78E-02 | 1.20E-02 |
|  |  | Bootstrap | 2.94E-05 | 2.19E-02 | 1.26E-02 |
| h2=0.7 | Bias | Analytical | 3.00E-08 | 8.91E-04 | 1.23E-03 |
|  |  | Approximated | 1.57E-05 | 5.23E-03 | 7.25E-03 |
|  |  | Bootstrap | 2.11E-05 | 3.71E-03 | 5.21E-03 |
|  | Dispersion | Analytical | 3.29E-05 | 4.16E-02 | 8.08E-02 |
|  |  | Approximated | 5.98E-05 | 4.63E-02 | 7.11E-02 |
|  |  | Bootstrap | 1.76E-04 | 6.45E-02 | 7.87E-02 |
|  | Reliability | Analytical | 2.71E-06 | 1.87E-02 | 8.21E-03 |
|  |  | Approximated | 3.94E-05 | 7.78E-03 | 2.28E-02 |
|  |  | Bootstrap | 5.06E-05 | 5.72E-03 | 3.39E-02 |
|  | Ratio of accuracies | Analytical |  | 1.35E-02 | 7.78E-03 |
|  |  | Bootstrap | 2.31E-05 | 1.60E-02 | 8.87E-03 |
|  | Predictivity | Analytical |  | 2.20E-02 | 1.53E-02 |
|  |  | Bootstrap | 4.10E-05 | 2.71E-02 | 1.59E-02 |
| h2=0.6 | Bias | Analytical | 4.11E-08 | 1.01E-03 | 1.22E-03 |
|  |  | Approximated | 1.17E-05 | 4.55E-03 | 6.35E-03 |
|  |  | Bootstrap | 1.60E-05 | 3.33E-03 | 4.67E-03 |
|  | Dispersion | Analytical | 3.46E-05 | 4.23E-02 | 7.87E-02 |
|  |  | Approximated | 8.23E-05 | 4.82E-02 | 6.81E-02 |
|  |  | Bootstrap | 1.77E-04 | 6.62E-02 | 7.69E-02 |
|  | Reliability | Analytical | 2.18E-06 | 1.66E-02 | 6.85E-03 |
|  |  | Approximated | 3.33E-05 | 6.56E-03 | 2.01E-02 |
|  |  | Bootstrap | 4.19E-05 | 4.94E-03 | 2.99E-02 |
|  | Ratio of accuracies | Analytical |  | 1.35E-02 | 7.88E-03 |
|  |  | Bootstrap | 2.69E-05 | 1.59E-02 | 9.16E-03 |
|  | Predictivity | Analytical |  | 2.75E-02 | 1.82E-02 |
|  |  | Bootstrap | 5.76E-05 | 3.41E-02 | 1.86E-02 |
| h2=0.5 | Bias | Analytical | 4.11E-08 | 1.03E-03 | 1.18E-03 |
|  |  | Approximated | 8.22E-06 | 3.78E-03 | 5.40E-03 |
|  |  | Bootstrap | 1.17E-05 | 2.81E-03 | 4.14E-03 |
|  | Dispersion | Analytical | 3.68E-05 | 4.20E-02 | 7.81E-02 |
|  |  | Approximated | 1.13E-04 | 4.92E-02 | 6.69E-02 |
|  |  | Bootstrap | 1.85E-04 | 6.76E-02 | 7.54E-02 |
|  | Reliability | Analytical | 1.60E-06 | 1.44E-02 | 5.82E-03 |
|  |  | Approximated | 2.74E-05 | 5.55E-03 | 1.77E-02 |
|  |  | Bootstrap | 3.39E-05 | 4.38E-03 | 2.63E-02 |
|  | Ratio of accuracies | Analytical |  | 1.36E-02 | 8.04E-03 |
|  |  | Bootstrap | 3.11E-05 | 1.60E-02 | 9.58E-03 |
|  | Predictivity | Analytical |  | 3.28E-02 | 2.20E-02 |
|  |  | Bootstrap | 8.31E-05 | 4.13E-02 | 2.13E-02 |
| h2=0.4 | Bias | Analytical | 4.56E-08 | 9.77E-04 | 1.10E-03 |
|  |  | Approximated | 5.34E-06 | 2.98E-03 | 4.48E-03 |
|  |  | Bootstrap | 7.92E-06 | 2.24E-03 | 3.56E-03 |
|  | Dispersion | Analytical | 4.14E-05 | 4.09E-02 | 7.67E-02 |
|  |  | Approximated | 1.53E-04 | 4.97E-02 | 6.57E-02 |
|  |  | Bootstrap | 2.06E-04 | 6.79E-02 | 7.53E-02 |
|  | Reliability | Analytical | 1.16E-06 | 1.19E-02 | 4.72E-03 |
|  |  | Approximated | 2.13E-05 | 4.49E-03 | 1.52E-02 |
|  |  | Bootstrap | 2.60E-05 | 3.73E-03 | 2.22E-02 |
|  | Ratio of accuracies | Analytical |  | 1.36E-02 | 8.40E-03 |
|  |  | Bootstrap | 3.65E-05 | 1.61E-02 | 1.02E-02 |
|  | Predictivity | Analytical |  | 3.80E-02 | 2.89E-02 |
|  |  | Bootstrap | 1.24E-04 | 4.98E-02 | 2.60E-02 |
| h2=0.3 | Bias | Analytical | 5.89E-08 | 8.99E-04 | 9.42E-04 |
|  |  | Approximated | 3.22E-06 | 2.27E-03 | 3.45E-03 |
|  |  | Bootstrap | 4.91E-06 | 1.70E-03 | 2.85E-03 |
|  | Dispersion | Analytical | 4.73E-05 | 4.04E-02 | 7.58E-02 |
|  |  | Approximated | 2.08E-04 | 5.10E-02 | 6.55E-02 |
|  |  | Bootstrap | 2.42E-04 | 6.94E-02 | 7.64E-02 |
|  | Reliability | Analytical | 7.40E-07 | 9.62E-03 | 3.47E-03 |
|  |  | Approximated | 1.58E-05 | 3.66E-03 | 1.20E-02 |
|  |  | Bootstrap | 1.88E-05 | 3.25E-03 | 1.75E-02 |
|  | Ratio of accuracies | Analytical |  | 1.39E-02 | 9.14E-03 |
|  |  | Bootstrap | 4.29E-05 | 1.65E-02 | 1.12E-02 |
|  | Predictivity | Analytical |  | 4.56E-02 | 4.20E-02 |
|  |  | Bootstrap | 1.99E-04 | 6.20E-02 | 3.57E-02 |
| h2=0.2 | Bias | Analytical | 5.56E-08 | 8.20E-04 | 8.16E-04 |
|  |  | Approximated | 1.60E-06 | 1.54E-03 | 2.45E-03 |
|  |  | Bootstrap | 2.50E-06 | 1.15E-03 | 2.17E-03 |
|  | Dispersion | Analytical | 5.58E-05 | 3.96E-02 | 7.61E-02 |
|  |  | Approximated | 2.90E-04 | 5.26E-02 | 6.74E-02 |
|  |  | Bootstrap | 3.07E-04 | 7.07E-02 | 8.00E-02 |
|  | Reliability | Analytical | 4.42E-07 | 7.18E-03 | 2.64E-03 |
|  |  | Approximated | 1.00E-05 | 2.91E-03 | 9.05E-03 |
|  |  | Bootstrap | 1.14E-05 | 2.77E-03 | 1.29E-02 |
|  | Ratio of accuracies | Analytical |  | 1.47E-02 | 9.86E-03 |
|  |  | Bootstrap | 5.12E-05 | 1.74E-02 | 1.22E-02 |
|  | Predictivity | Analytical |  | 5.83E-02 | 6.60E-02 |
|  |  | Bootstrap | 3.85E-04 | 8.45E-02 | 5.45E-02 |
| h2=0.1 | Bias | Analytical | 5.00E-08 | 6.87E-04 | 6.37E-04 |
|  |  | Approximated | 5.43E-07 | 7.90E-04 | 1.42E-03 |
|  |  | Bootstrap | 8.51E-07 | 6.14E-04 | 1.42E-03 |
|  | Dispersion | Analytical | 7.01E-05 | 3.80E-02 | 7.63E-02 |
|  |  | Approximated | 4.41E-04 | 5.64E-02 | 7.44E-02 |
|  |  | Bootstrap | 4.45E-04 | 7.38E-02 | 9.12E-02 |
|  | Reliability | Analytical | 1.98E-07 | 4.18E-03 | 1.58E-03 |
|  |  | Approximated | 4.02E-06 | 1.97E-03 | 5.17E-03 |
|  |  | Bootstrap | 4.42E-06 | 1.98E-03 | 7.20E-03 |
|  | Ratio of accuracies | Analytical |  | 1.56E-02 | 9.78E-03 |
|  |  | Bootstrap | 6.26E-05 | 1.86E-02 | 1.21E-02 |
|  | Predictivity | Analytical |  | 1.05E-01 | 1.49E-01 |
|  |  | Bootstrap | 1.21E-03 | 1.63E-01 | 1.14E-01 |

**Supplementary Table 2.** Average squared differences between estimated and true variance, lower bound of the 95% confidence interval (lCI), and upper bound of the 95% confidence interval (uCI) for Example 1 for different proportions of phenotyped animals.

|  |  |  | Var | lCI | uCI |
| --- | --- | --- | --- | --- | --- |
| p=0.9 | Bias | Analytical | 6.00E-08 | 3.06E-03 | 7.24E-04 |
|  |  | Approximated | 2.01E-06 | 8.61E-03 | 2.19E-03 |
|  |  | Bootstrap | 2.40E-06 | 5.91E-03 | 1.08E-03 |
|  | Dispersion | Analytical | 3.46E-05 | 5.60E-03 | 6.88E-02 |
|  |  | Approximated | 1.09E-04 | 5.44E-02 | 9.69E-03 |
|  |  | Bootstrap | 6.98E-05 | 5.14E-02 | 1.45E-02 |
|  | Reliability | Analytical | 4.42E-05 | 1.10E-01 | 9.06E-03 |
|  |  | Approximated | 1.66E-04 | 5.45E-03 | 1.08E-01 |
|  |  | Bootstrap | 2.06E-04 | 7.93E-03 | 1.60E-01 |
|  | Ratio of accuracies | Analytical |  | 1.09E-02 | 1.17E-02 |
|  |  | Bootstrap | 3.56E-05 | 1.55E-02 | 1.73E-02 |
|  | Predictivity | Analytical | 9.33E-04 | 1.57E-02 | 1.66E-02 |
|  |  | Bootstrap | 5.76E-05 | 5.29E-03 | 1.03E-02 |
| p=0.8 | Bias | Analytical | 2.00E-07 | 1.09E-02 | 6.60E-03 |
|  |  | Approximated | 2.51E-06 | 1.71E-02 | 8.20E-03 |
|  |  | Bootstrap | 2.59E-06 | 1.15E-02 | 1.00E-02 |
|  | Dispersion | Analytical | 5.00E-07 | 4.68E-02 | 1.19E-04 |
|  |  | Approximated | 2.94E-04 | 1.19E-02 | 5.51E-02 |
|  |  | Bootstrap | 1.80E-04 | 1.32E-02 | 4.27E-02 |
|  | Reliability | Analytical | 8.70E-07 | 1.72E-01 | 4.99E-02 |
|  |  | Approximated | 3.73E-04 | 1.99E-02 | 2.65E-01 |
|  |  | Bootstrap | 4.46E-04 | 8.29E-03 | 3.53E-01 |
|  | Ratio of accuracies | Analytical |  | 1.74E-02 | 4.23E-02 |
|  |  | Bootstrap | 3.07E-05 | 1.20E-02 | 5.47E-02 |
|  | Predictivity | Analytical |  | 1.49E-01 | 5.25E-02 |
|  |  | Bootstrap | 1.46E-03 | 6.95E-02 | 4.69E-03 |
| p=0.7 | Bias | Analytical | 1.45E-06 | 5.25E-03 | 5.16E-03 |
|  |  | Approximated | 3.72E-06 | 4.40E-03 | 1.40E-02 |
|  |  | Bootstrap | 3.63E-06 | 8.43E-03 | 8.17E-03 |
|  | Dispersion | Analytical | 2.37E-04 | 1.55E-01 | 6.38E-02 |
|  |  | Approximated | 1.21E-03 | 3.55E-02 | 2.39E-01 |
|  |  | Bootstrap | 1.02E-03 | 2.87E-02 | 2.47E-01 |
|  | Reliability | Analytical | 1.49E-06 | 1.85E-01 | 5.73E-02 |
|  |  | Approximated | 3.95E-04 | 2.17E-02 | 2.79E-01 |
|  |  | Bootstrap | 4.86E-04 | 7.02E-03 | 3.81E-01 |
|  | Ratio of accuracies | Analytical |  | 2.24E-03 | 8.15E-02 |
|  |  | Bootstrap | 7.76E-05 | 8.29E-04 | 9.35E-02 |
|  | Predictivity | Analytical |  | 8.45E-02 | 9.47E-02 |
|  |  | Bootstrap | 6.39E-04 | 4.00E-02 | 1.51E-01 |
| p=0.6 | Bias | Analytical | 2.10E-07 | 1.95E-03 | 2.00E-02 |
|  |  | Approximated | 5.30E-06 | 2.00E-03 | 3.28E-02 |
|  |  | Bootstrap | 5.10E-06 | 2.68E-03 | 2.19E-02 |
|  | Dispersion | Analytical | 9.49E-06 | 4.06E-01 | 1.25E-01 |
|  |  | Approximated | 3.77E-04 | 1.54E-01 | 3.66E-01 |
|  |  | Bootstrap | 1.74E-04 | 2.02E-01 | 3.05E-01 |
|  | Reliability | Analytical | 3.26E-06 | 3.60E-01 | 1.16E-01 |
|  |  | Approximated | 3.76E-04 | 1.07E-01 | 3.77E-01 |
|  |  | Bootstrap | 5.24E-04 | 3.99E-02 | 5.53E-01 |
|  | Ratio of accuracies | Analytical |  | 2.51E-01 | 2.90E-01 |
|  |  | Bootstrap | 6.62E-05 | 1.93E-01 | 3.44E-01 |
|  | Predictivity | Analytical |  | 3.75E-01 | 1.50E-01 |
|  |  | Bootstrap | 1.17E-04 | 2.73E-01 | 2.05E-01 |
| p=0.5 | Bias | Analytical | 2.30E-07 | 1.07E-03 | 8.66E-04 |
|  |  | Approximated | 6.28E-06 | 6.02E-03 | 1.09E-02 |
|  |  | Bootstrap | 2.95E-06 | 5.62E-04 | 3.81E-03 |
|  | Dispersion | Analytical | 4.90E-05 | 7.36E-03 | 2.49E-02 |
|  |  | Approximated | 1.00E-03 | 9.21E-02 | 1.41E-02 |
|  |  | Bootstrap | 1.12E-03 | 1.53E-01 | 3.98E-02 |
|  | Reliability | Analytical | 7.40E-07 | 2.79E-02 | 8.28E-03 |
|  |  | Approximated | 2.13E-04 | 3.45E-02 | 4.14E-02 |
|  |  | Bootstrap | 2.70E-04 | 4.85E-02 | 7.72E-02 |
|  | Ratio of accuracies | Analytical |  | 1.33E-01 | 1.38E-03 |
|  |  | Bootstrap | 2.58E-04 | 1.71E-01 | 2.38E-03 |
|  | Predictivity | Analytical |  | 2.03E-01 | 4.46E-02 |
|  |  | Bootstrap | 1.21E-03 | 3.34E-01 | 2.24E-02 |
| p=0.4 | Bias | Analytical | 1.40E-07 | 2.09E-02 | 4.55E-03 |
|  |  | Approximated | 2.40E-05 | 6.00E-02 | 9.11E-03 |
|  |  | Bootstrap | 2.55E-05 | 4.08E-02 | 6.39E-03 |
|  | Dispersion | Analytical | 4.10E-04 | 1.43E-01 | 1.51E-01 |
|  |  | Approximated | 2.38E-03 | 3.97E-01 | 6.27E-02 |
|  |  | Bootstrap | 3.27E-03 | 6.46E-01 | 3.94E-02 |
|  | Reliability | Analytical | 6.69E-05 | 2.30E-02 | 2.22E-02 |
|  |  | Approximated | 4.76E-04 | 1.17E-01 | 8.99E-03 |
|  |  | Bootstrap | 4.40E-04 | 1.35E-01 | 1.48E-02 |
|  | Ratio of accuracies | Analytical |  | 1.04E-01 | 1.03E-02 |
|  |  | Bootstrap | 1.25E-04 | 1.31E-01 | 7.40E-03 |
|  | Predictivity | Analytical |  | 3.33E-01 | 1.34E-01 |
|  |  | Bootstrap | 7.11E-03 | 5.99E-01 | 4.73E-02 |
| p=0.3 | Bias | Analytical | 6.50E-07 | 2.24E-02 | 8.21E-03 |
|  |  | Approximated | 4.98E-05 | 9.38E-02 | 8.73E-03 |
|  |  | Bootstrap | 6.84E-05 | 7.62E-02 | 4.17E-03 |
|  | Dispersion | Analytical | 1.53E-04 | 4.69E-01 | 1.78E-01 |
|  |  | Approximated | 1.81E-03 | 2.08E-01 | 4.69E-01 |
|  |  | Bootstrap | 1.84E-03 | 2.42E-01 | 5.06E-01 |
|  | Reliability | Analytical | 4.56E-06 | 2.02E-01 | 6.10E-02 |
|  |  | Approximated | 1.37E-04 | 7.22E-02 | 1.75E-01 |
|  |  | Bootstrap | 2.81E-04 | 1.77E-02 | 3.15E-01 |
|  | Ratio of accuracies | Analytical |  | 1.76E-01 | 1.56E-01 |
|  |  | Bootstrap | 1.67E-04 | 1.06E-01 | 2.22E-01 |
|  | Predictivity | Analytical |  | 5.94E-01 | 2.11E-01 |
|  |  | Bootstrap | 3.58E-04 | 2.75E-01 | 4.76E-01 |
| p=0.2 | Bias | Analytical | 1.80E-07 | 3.99E-05 | 9.45E-03 |
|  |  | Approximated | 1.46E-04 | 4.38E-02 | 9.24E-02 |
|  |  | Bootstrap | 1.50E-04 | 2.21E-02 | 5.66E-02 |
|  | Dispersion | Analytical | 8.33E-04 | 4.09E-01 | 9.42E-01 |
|  |  | Approximated | 4.31E-03 | 6.95E-01 | 6.04E-01 |
|  |  | Bootstrap | 5.66E-03 | 1.04E+00 | 3.09E-01 |
|  | Reliability | Analytical | 3.29E-06 | 4.06E-02 | 1.42E-01 |
|  |  | Approximated | 2.94E-05 | 9.20E-02 | 7.79E-02 |
|  |  | Bootstrap | 9.73E-06 | 9.64E-02 | 7.77E-02 |
|  | Ratio of accuracies | Analytical |  | 3.78E-01 | 4.99E-02 |
|  |  | Bootstrap | 7.55E-04 | 4.36E-01 | 4.81E-02 |
|  | Predictivity | Analytical |  | 1.13E+00 | 1.18E+00 |
|  |  | Bootstrap | 2.13E-03 | 1.50E+00 | 1.05E+00 |
| p=0.1 | Bias | Analytical | 1.80E-07 | 6.49E-03 | 3.15E-02 |
|  |  | Approximated | 5.97E-04 | 6.43E-02 | 2.59E-01 |
|  |  | Bootstrap | 8.60E-04 | 4.59E-02 | 2.15E-01 |
|  | Dispersion | Analytical | 1.73E-03 | 1.65E+00 | 4.86E+00 |
|  |  | Approximated | 1.31E-03 | 2.32E+00 | 3.89E+00 |
|  |  | Bootstrap | 5.63E-03 | 3.06E+00 | 4.99E+00 |
|  | Reliability | Analytical | 1.56E-05 | 7.75E-03 | 4.45E-03 |
|  |  | Approximated | 5.09E-05 | 1.22E-03 | 1.38E-02 |
|  |  | Bootstrap | 1.59E-04 | 1.07E-02 | 6.23E-02 |
|  | Ratio of accuracies | Analytical |  | 4.36E-02 | 3.82E-02 |
|  |  | Bootstrap | 1.29E-03 | 2.62E-01 | 1.71E-02 |
|  | Predictivity | Analytical |  | 3.63E-01 | 1.38E+00 |
|  |  | Bootstrap | 7.70E-03 | 1.41E+00 | 7.23E-01 |
